# Characterization of the β-tubulin gene family in *Ascaris lumbricoides* and *Ascaris suum* and its implication for the molecular detection of benzimidazole resistance

**DOI:** 10.1371/journal.pntd.0009777

**Published:** 2021-09-27

**Authors:** Sara Roose, Russell W. Avramenko, Stephen M. J. Pollo, James D. Wasmuth, Shaali Ame, Mio Ayana, Martha Betson, Piet Cools, Daniel Dana, Ben P. Jones, Zeleke Mekonnen, Arianna Morosetti, Abhinaya Venkatesan, Johnny Vlaminck, Matthew L. Workentine, Bruno Levecke, John S. Gilleard, Peter Geldhof

**Affiliations:** 1 Department of Virology, Parasitology and Immunology, Ghent University, Merelbeke, Belgium; 2 Department of Comparative Biology and Experimental Medicine, Faculty of Veterinary Medicine, Host-Parasite Interactions (HPI) Research Training Network, University of Calgary, Calgary, Alberta, Canada; 3 Department of Ecosystem and Public Health, Faculty of Veterinary Medicine, Host-Parasite Interactions (HPI) Research Training Network, University of Calgary, Calgary, Alberta, Canada; 4 Public Health Laboratory–Ivo de Carneri, Chake Chake, United Republic of Tanzania; 5 Institute of Health, Faculty of Health Science, School of Medical Laboratory Science, Jimma University, Jimma, Ethiopia; 6 Department of Veterinary Epidemiology and Public Health, University of Surrey, Guildford, Surrey, United Kingdom; The University of Melbourne, AUSTRALIA

## Abstract

**Background:**

The treatment coverage of control programs providing benzimidazole (BZ) drugs to eliminate the morbidity caused by soil-transmitted helminths (STHs) is unprecedently high. This high drug pressure may result in the development of BZ resistance in STHs and so there is an urgent need for surveillance systems detecting molecular markers associated with BZ resistance. A critical prerequisite to develop such systems is an understanding of the gene family encoding β-tubulin proteins, the principal targets of BZ drugs.

**Methodology and principal findings:**

First, the β-tubulin gene families of *Ascaris lumbricoides* and *Ascaris suum* were characterized through the analysis of published genomes. Second, RNA-seq and RT-PCR analyses on cDNA were applied to determine the transcription profiles of the different gene family members. The results revealed that *Ascaris* species have at least seven different β-tubulin genes of which two are highly expressed during the entire lifecycle. Third, deep amplicon sequencing was performed on these two genes in more than 200 adult *A*. *lumbricoides* (Ethiopia and Tanzania) and *A*. *suum* (Belgium) worms, to investigate the intra- and inter-species genetic diversity and the presence of single nucleotide polymorphisms (SNPs) that are associated with BZ resistance in other helminth species; F167Y (TTC>TAC or TTT>TAT), E198A (GAA>GCA or GAG>GCG), E198L (GAA>TTA) and F200Y (TTC>TAC or TTT>TAT). These particular SNPs were absent in the two investigated genes in all three *Ascaris* populations.

**Significance:**

This study demonstrated the presence of at least seven β-tubulin genes in *Ascaris* worms. A new nomenclature was proposed and prioritization of genes for future BZ resistance research was discussed. This is the first comprehensive description of the β-tubulin gene family in *Ascaris* and provides a framework to investigate the prevalence and potential role of β-tubulin sequence polymorphisms in BZ resistance in a more systematic manner than previously possible.

## Author summary

Benzimidazole (BZ) drugs remain the standard of treatment in large-scale deworming programs that aim to control the morbidity caused by intestinal worms. As these deworming programs are expanding world-wide, there is an increasing risk of worms becoming resistant to BZ drugs, highlighting the necessity for tools to detect gene mutations associated with drug resistance. However, the development of such tools is impeded by a lack of insights into the genes that are coding for β-tubulin proteins, which are the principal targets of BZ drugs. The aim of this study was to comprehensively characterize these genes in the worm species *Ascaris lumbricoides* and *Ascaris suum*. The findings highlight that these species have at least seven β-tubulin genes. Only two genes are highly expressed throughout the different life stages of the worm, and hence are more likely to be involved in the development of BZ resistance. No mutations that have previously been associated with BZ resistance in other intestinal worms were found. This study provides a baseline towards more efficient and accurate monitoring of drug resistance in large-scale deworming programs.

## Introduction

The latest global reports on control programs for soil-transmitted helminths (STHs; *Ascaris lumbricoides*, *Trichuris trichiura*, *Necator americanus* and *Ancylostoma duodenale*) show that drug coverage continues to rise. In 2019, the benzimidazole (BZ) drugs albendazole (ALB) and mebendazole (MEB) were administered to 777.5 million people worldwide, which covered 58.64% of all (pre-)school-aged children ((pre-)SAC) in need of treatment (in 2010 this was only 30.94%) [1,2]. It is anticipated that this number will continue to increase since the target of the World Health Organization (WHO) is to reduce moderate-to-heavy intensity infection prevalence to less than 2% by 2030 [3]. As demonstrated repeatedly in animal STHs, the world-wide upscale in drug distribution increases the risk for the development of anthelmintic resistance, highlighting the necessity for tools to detect mutations in the genes that are encoding for β-tubulin proteins, the principal targets of BZ drugs [4,5]. A number of single nucleotide polymorphisms (SNPs) in β-tubulin genes (F167Y (TTC>TAC or TTT>TAT), E198A (GAA>GCA or GAG>GCG), E198L (GAA>TTA) and F200Y (TTC>TAC or TTT>TAT)) are associated with BZ resistance in a variety of animal STHs (e.g. *Haemonchus contortus*, *Teladorsagia circumcincta* and *Ancylostoma caninum* [6–24]) and conferred phenotypic resistance in the transgenic model organism *Caenorhabditis elegans* [25]. However, the number of β-tubulin genes and their relationships vary between nematode species, and so care needs to be taken when extrapolating information between species, particularly those that are more distantly related [15,26,27]. For *H*. *contortus*, which has four β-tubulin genes, the F167Y (TTC>TAC or TTT>TAT), E198A (GAA>GCA or GAG>GCG) and F200Y (TTC>TAC or TTT>TAT) resistance mutations have been detected in one β-tubulin gene (*Hco-tbb-iso-1*), often at high frequency in BZ resistant populations [10,17,19–24,28]. However, there is some evidence that also a second β-tubulin gene (*Hco-tbb-iso-2*) has a role in BZ resistance, although possibly less important [24,29]. The two remaining *H*. *contortus* β-tubulin genes (*Hco-tbb-iso-3* and *Hco-tbb-iso-4*) are expressed at extremely low levels and in a restricted spatial manner and so are unlikely to have a role in resistance [27]. Experimental data for *C*. *elegans*, which has six β-tubulin genes, has shown that all BZ resistance mutations generated by chemical mutagenesis map to a single gene, namely *Cel-ben-1* [27,30]. But there is evidence that BZ resistance in natural strains of *C*. *elegans* is not only associated with the loss of one β-tubulin gene and that there are multiple mechanisms underlying BZ resistance, involving multiple loci [31,32]. Although *H*. *contortus* and *C*. *elegans* are relatively closely related, both belonging to Clade V in the nematode phylogeny [33], the genes involved in BZ resistance do not share one-to-one orthology [27]. The complex β-tubulin phylogeny thereby not only complicates comparative analysis into the potential involvement of genes in BZ resistance but also makes a common nomenclature for the β-tubulin genes across species difficult. Today, little is known about the composition of the β-tubulin gene family in human STH species. Krücken and colleagues investigated four β-tubulin genes in *A*. *lumbricoides* [34], but remaining studies have focused on a single gene to explore the presence of SNPs that may be potentially associated with BZ resistance in human *A*. *lumbricoides* [35–41] and porcine *Ascaris suum* [42] populations. In *A*. *lumbricoides*, a reduced efficacy to BZ has been reported but no SNPs were found in the four investigated β-tubulin genes [34]. Other studies detected the SNP TTC>TAC in codon 167 but the mutation was not associated with reduced efficacy [35,37]. In *A*. *suum*, to date none of the BZ resistance associated SNPs have been described in the single β-tubulin gene that has been examined [42]. Consequently, for both species, the current evidence for these SNPs as a marker for BZ resistance is not yet well-defined [4,43]. Furthermore, because of the poor knowledge of the β-tubulin gene family composition in the *Ascaris* species, it is difficult to interpret the relevance of the absence of potential resistance conferring SNPs in a particular β-tubulin gene without knowledge of other β-tubulin loci that may be relevant.

The overall aim of the present study was to comprehensively characterize the β-tubulin gene family in *A*. *lumbricoides* and *A*. *suum*, both in Clade III of the nematode phylogeny, with the goal of prioritizing the most relevant genes in the context of BZ resistance. The porcine parasite *A*. *suum* is phylogenetically very closely related to *A*. *lumbricoides*; they have recently been shown to be an interbreeding species complex [44]. For *A*. *suum* a high-quality genome assembly and transcriptome is available and worm material is accessible through experimental infection of pigs. Moreover, BZ drugs have already been used for decades to combat *Ascaris* infections in the pig industry, further highlighting the potential of *A*. *suum* as an excellent experimental model with respect to BZ resistance research.

## Methods

### Ethics statement

*A*. *lumbricoides* worms were collected during two worm expulsion studies as part of the Starworms project. Ethical approval to conduct the expulsion studies was obtained from the Institutional Review Board (IRB) of the Faculty of Medicine and Health Sciences (Ghent University) and Ghent University Hospital (Belgium; reference number: B670201837418), The Zanzibar Medical Research and Ethics Committee (Tanzania; reference number: ZAHREC/03/DEC/2018) and Jimma University (Ethiopia; reference number: IHRPG/269/2018). Parent(s)/guardian(s) of participants signed an informed consent document indicating that they understood the purpose and procedures of the study, and that they allowed their child to participate. Children younger than 12 years of age had to orally assent in order to participate, participants of 12 years of age or older were only included if they gave written consent.

### Research workflow

**Fig 1:**
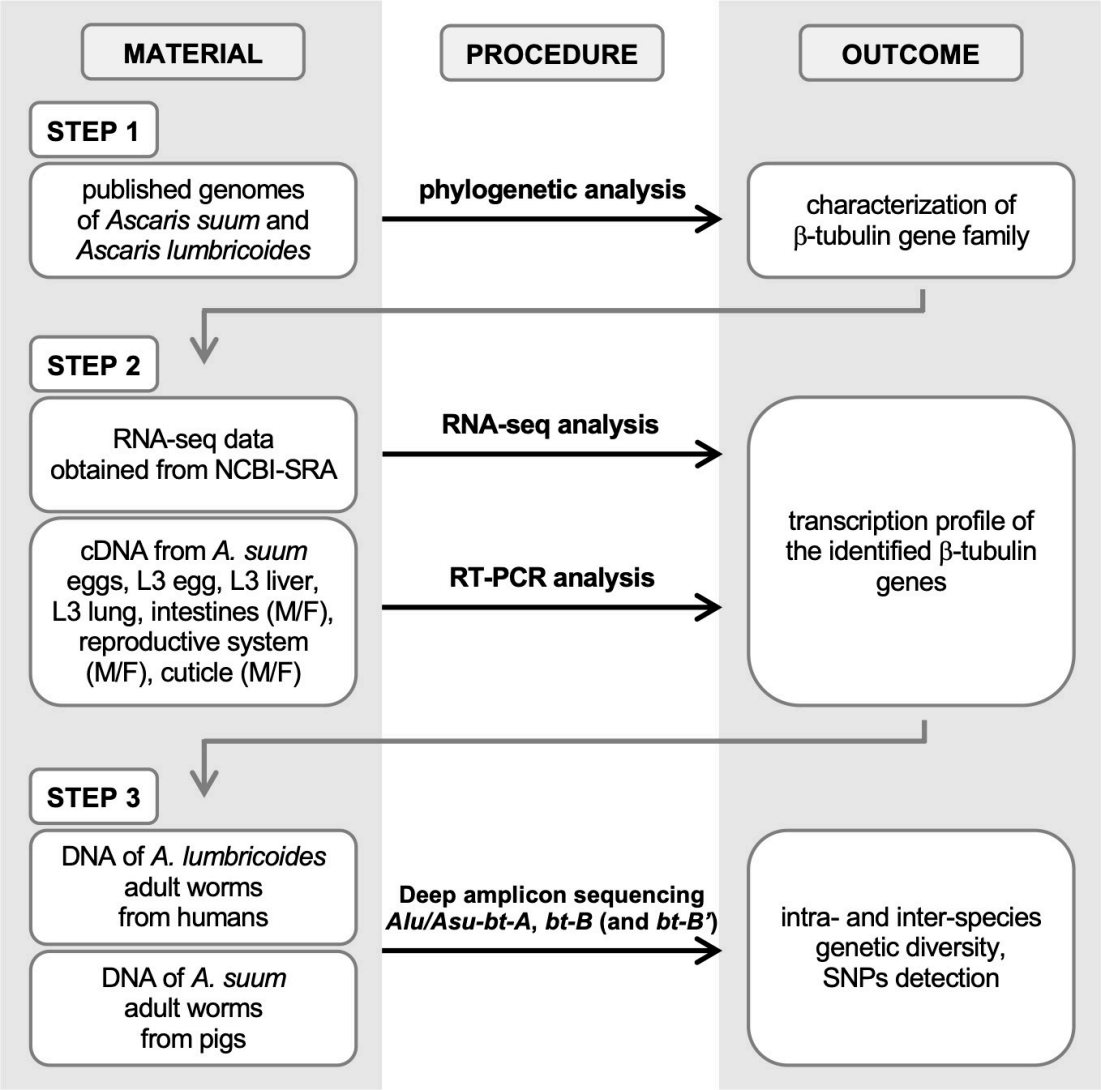
Schematic representation of the research workflow. RNA-Seq: RNA sequencing, NCBI-SRA: NCBI Sequence Read Archive, cDNA: complementary DNA, L3: third larval stage, M: male, F: female, RT-PCR: reverse transcription PCR, SNPs: single nucleotide polymorphisms.

We performed a series of experiments that were organized in three consecutive steps (summarized in **Fig 1**). In a first step, we characterized and compared the β-tubulin gene families of *A*. *lumbricoides* and *A*. *suum*. In a second step, we analyzed the transcription profile of the different β-tubulin genes during the life cycle of *A*. *suum*. In a third step, we assessed the intra- and inter-species genetic diversity and presence of known BZ resistance associated SNPs in β-tubulin genes in both human and porcine BZ-drug-exposed worm populations applying a deep amplicon sequencing approach. This final step was applied on strategically selected genes based on the outcome of steps 1 and 2.

### *Ascaris* material

Messenger RNA from *A*. *suum* eggs, infectious stage 3 larvae (L3) from eggs, migrating L3 from liver and lungs, as well as cuticle, intestinal, and reproductive tissues from adult male and female *A*. *suum* worms was purified by Wang and colleagues [45]. Complementary DNA (cDNA) was synthesized using the iScript cDNA synthesis kit (Bio-rad) following the manufacturer’s instructions.

Genomic DNA was extracted from adult *Ascaris* worms for which the origin and method of collection are summarized in **Table 1**. Human *A*. *lumbricoides* worms were collected during two worm expulsion studies. The human study population consisted of school children of 7 to 14 years of age, living in two endemic regions, i.e. Pemba Island (Tanzania) and Jimma Town (Ethiopia). A single dose of 400 mg ALB (GlaxoSmithKline) was orally administered to the children for one day (Tanzania) or three consecutive days (Ethiopia). Expelled worms were collected from stool from the past 24 hours for seven consecutive days. As indicated in **Table 1**, a total of 106 *A*. *lumbricoides* worms were analyzed, including 29 worms from 7 Ethiopian children and 77 worms from 53 Tanzanian children. The 109 *A*. *suum* worms were collected from porcine intestines obtained at different slaughterhouses in Flanders. The random collection of both male and female worms was done at three different time points within a six-month period. This time interval assured a set of worms coming from different farms.

**Table 1:** Overview of origin of the adult *Ascaris* worms used for deep amplicon sequencing.

Origin	Study site	Study design	Number of worms	Number of subjects	Median (range) of worms per subject
Human	Ethiopia (Jimma Town)	Expulsion within 7 days after treatment with 400 mg ALB for 3 consecutive days	29	7	3 (1–7)
Human	Tanzania (Pemba Island)	Expulsion within 7 days after single treatment with 400 mg ALB	77	53	1 (1–7)
Porcine	Belgium (Flanders)	Collection from intestines after slaughter	109	NA	NA

Anterior sections (0.5–1 cm) of both *A*. *lumbricoides* and *A*. *suum* adult worms were individually lysed in 300 μL Buffer ATL (Qiagen) and 50 μL Proteinase K (Qiagen) for 24h to 48h at 55°C under gentle agitation (300 rpm). One volume of phenol-chloroform (1:1) (Sigma) was added, followed by centrifugation for 10 min at 10,000 g. The supernatant was recovered and after addition of 3 M sodium acetate (1:10) (Sigma) and isopropanol (1:1), single worm DNA was precipitated by centrifugation for 10 min at 16,000 g. The pellet was washed twice with 80% ethanol and eluted in 50 μL molecular-grade water. DNA concentration was measured with the Nanodrop 2000 (ThermoFisher Scientific).

### Characterization and comparison of the β-tubulin gene families

Protein sequences available at WormBase ParaSite from *A*. *lumbricoides* (PRJEB4950) and *A*. *suum* (PRJNA80881) were used as query sequences in a BLASTP search against a database of *C*. *elegans* proteins (PRJNA13758) [46–48]. *Ascaris* proteins whose top hit was a protein from one of the six β-tubulin genes from *C*. *elegans* (B0272.1, C36E8.5, C54C6.2, K01G5.7, T04H1.9, ZK154.3), were considered candidate β-tubulin genes. The alignments were manually inspected to ensure that the *Ascaris* proteins were at least 400 amino acids in length. All candidate proteins were searched against the Pfam database to ensure that they contain the ‘Tubulin/FtsZ family, GTPase’ domain (PF00091) and the ‘Tubulin C-terminal’ domain (PF03953) [49]. This revealed that the protein with accession GS_10401 contained the N terminus of the ‘Tubulin GTPase’ domain, while the protein with accession GS_22804 contained the rest of that domain and the ‘Tubulin C-terminal’ domain. As their genes are adjacent in the genome assembly, we considered this a mistake in the original annotation and decided to merge the two to give one protein sequence.

The β-tubulin genes identified in *Ascaris* species (Clade III in nematode phylogeny) were aligned to those of the other three human STH species (*T*. *trichiura* (PRJEB535) belonging to Clade I, *N*. *americanus* (PRJNA72135) and *Ancylostoma ceylanicum* (PRJNA231479) belonging to Clade V), and to the well characterized β-tubulin families of *H*. *contortus* (PRJEB506) and *C*. *elegans* (PRJNA13758) (both Clade V) [30,50]. Additionally, two other members of Clade III, and thus more closely related to *Ascaris*, were included in the analysis: *Parascaris equorum* (equines) and *Ascaridia galli* (poultry) for which sequences published by Tyden and colleagues were used [26]. Gene ID and Transcript ID of all protein sequences covered in the analysis are provided in **S1 Info**, including accession numbers of source worm genomes. The genome assembly and annotation of *A*. *ceylanicum* were used as close representatives of *A*. *duodenale*, since the latter genome assembly has low quality metrics [46,47]. β-tubulin gene names were adopted from published literature and genes that have not been named yet were indicated by their accession number.

Protein sequences were aligned using MUSCLE (maximum number of iterations: 8; other parameters: default) [51]. The alignment was viewed in Geneious (v10.2.6; https://geneious.com) and alignment positions with greater than 10% gaps from the phylogenetic reconstructions were masked. Two sequences, TBB-6 from *C*. *elegans* and Ttr-TTRE_0000019101 from *T*. *trichiura*, were noticeably divergent from other sequences in the alignment and in the preliminary phylogenetic reconstructions confounded the topology, likely due to long-branch attraction, so were removed from subsequent analyses [52]. For phylogenetics, three β-tubulins from *Drosophila melanogaster* were included to serve as an outgroup. RAxML (version 8) was used to generate maximum likelihood-based trees (protein model: GAMMA LG; algorithm: rapid bootstrapping; replicates: 1,000) [53]. MRBAYES (version 3.2.6) was used to reconstruct a Bayesian phylogeny (rate matrix: poisson; rate variation: gamma; chain length: 1,100,000; subsampling frequency: 200; heated chains: 4; burn-in length: 100,000; heated chain temp: 0.2) [54]. The phylogenetic figures were viewed and further annotated in iTOL (version 5.7) [55] and Adobe Illustrator (https://adobe.com/products/illustrator).

Previously investigated β-tubulin genes from eight papers studying these genes in *A*. *lumbricoides* [15,34,35,37–41] and three papers in *A*. *suum* [15,34,42] were BLAST searched against the *Ascaris* genes identified in this study to determine which β-tubulin gene(s) were examined in each of the previous studies [48].

### Transcription profiles of the β-tubulin genes during the *Ascaris* life cycle

Gene specific primers were designed using Primer3 software (http://bioinfo.ut.ee/primer3/), based on the coding sequences of the β-tubulin genes, retrieved from the *A*. *suum* published genome (PRJNA62057) available at WormBase ParaSite [46,47]. The full list of forward and reverse primers can be found in **S2 Info**. The actin gene was used as a control gene (CB039781). Actin primers were adopted from Vlaminck et al., 2011 [56]. To check gene specificity, all primers were searched using BLAST against the published *A*. *suum* and *A*. *lumbricoides* genomes (PRJNA62057, PRJNA80881 and PRJEB4950) [46,47]. Reverse transcription (RT)-PCR reactions were performed on cDNA from worm eggs, L3 larvae, and cuticle, intestinal, and reproductive tissues from adult male and female *A*. *suum* worms under the following conditions: 1X Green GoTaq Flexi Buffer (Promega), 2 mM MgCl_2_, 0.2 mM dNTPs, 0.25 μM gene specific primer forward, 0.25 μM gene specific primer reverse, 1.25 U GoTaq G2 DNA Polymerase (Promega) and 250–500 ng of cDNA. The thermocycling parameters were 95°C for 2 min, followed by 35 cycles of 95°C for 30 s, 54°C for 30 s, 72°C for 1 min, and a final extension of 72°C for 10 min. Aliquots (5 μL) of individual RT-PCR products were run electrophoretically on 1.5% agarose gels stained with ethidium bromide (0.4 μg/ml) (Sigma-Aldrich). Results were visualized with the Bio-Rad Gel Doc EZ Imager (Bio-Rad Laboratories nv.) by ultraviolet trans-illumination, and fragment sizes determined by comparison with a 100 bp DNA ladder (Promega).

*A*. *suum* RNA-seq reads were downloaded from the NCBI Sequence Read Archive (SRA) from SRA studies SRP013573 [57], SRP013609 [57], SRP005511 [58] and SRP010159 [59] (**S3 Info**). *A*. *suum* transcripts were downloaded from WormBase ParaSite (PRJNA80881) [46,47] and the transcripts for the β-tubulins were renamed and joined to match the identified β-tubulin genes. RNA-seq reads were then pseudo-mapped using kallisto v0.46.2 [60] to the modified *A*. *suum* transcripts to obtain read counts and transcripts per million (TPM) expression values for every transcript. For each sample type, the mean TPM of each β-tubulin was calculated and the proportion of total β-tubulin expression represented by each β-tubulin gene in each sample type was plotted.

### Intra- and inter-species genetic diversity and presence of candidate BZ resistance associated SNPs in drug-exposed adult *A*. *lumbricoides* and *A*. *suum* worm populations

To amplify a 519 bp fragment of *Alu/Asu-bt-A* (ALUE_0000927201, GS_23993), a single forward primer (BtA-For) and a single reverse primer (BtA-Rev) were designed for both *A*. *lumbricoides* and *A*. *suum*. For a 689 bp fragment of *Alu/Asu-bt-B* (ALUE_0000986501, GS_01240) a single forward primer (BtB-For), but two different reverse primers (BtB-Rev1 and BtB-Rev2) were created to ensure amplification of *Asu-bt-B* in *A*. *suum*, and both *Alu-bt-B* and *Alu-bt-B’ (*ALUE_0001827701) in *A*. *lumbricoides* for which it was not clear whether these were allelic variants or separate genes. All primers were designed using Geneious (v10.2.6; https://geneious.com). The locus specific primers were adapted for Illumina deep sequencing as described by Avramenko et al. [61]. A complete list of all adapted primers can be found in **S4 Info**. In general, the primer design was as follows: 5’–Illumina adapter sequence– 0 to 3 random nucleotides–locus specific primer– 3’. Four forward adapter primers, and four or eight reverse adapter primers were mixed in equal concentrations and used for the first round of PCR under the following conditions: 1X KAPA HiFi HotStart Fidelity Buffer (KAPA Biosystems), 0.3 μM forward primer mix, 0.3 μM reverse primer mix, 0.2 mM dNTPs, 0.5 U KAPA HiFi HotStart Polymerase (KAPA Biosystems), 2 μg bovine serum albumin (BSA) and a 1:250 dilution of single worm DNA (variable concentration). The thermocycling parameters were 95°C for 3 min, followed by 35 cycles of 98°C for 20 s, 61°C for 15 s, 72°C for 30 s, and a final extension of 72°C for 2 min. PCR products were purified with AMPure XP Magnetic Beads (1X) (Beckman Coulter Inc.) following the manufacturer’s recommended protocol.

Illumina barcode indices and P5/P7 sequencing regions were added to the amplicons by limited cycle PCR with primers of the Nextera XT Index Kit v2 set (Illumina Inc.). All primer sequences are provided in **S4 Info**. The PCR was performed as described by Avramenko et al. [61] with 5 μL of first-round clean PCR product as template. Amplicons were purified with AMPure XP Magnetic Beads (1X) (Beckman Coulter Inc.) following the manufacturer’s recommended protocol.

The concentration of the second-round clean PCR product was measured using the Implen NanoPhotometer NP80 and 50 ng of each sample was pooled to produce a master sequencing library. The final concentration of this pooled library was assessed with the KAPA qPCR Library Quantification Kit (KAPA Biosystems), following the manufacturer’s recommended protocol. The prepared pooled library was run on an Illumina MiSeq Desktop Sequencer using a 600-cycle MiSeq Reagent Kit v3 (Illumina Inc., MS-102-3003) at a concentration of 15 pM with the addition of 20% PhiX Control v3 (Illumina Inc., FC-110-3001). The MiSeq was set to generate only FASTQ files with no post-run analysis. Based on their supplied index combinations, samples were automatically demultiplexed by the MiSeq. All protocols were carried out per Illumina’s standard MiSeq operating protocol (Illumina Inc.).

Raw FASTQ files generated were analyzed with the DADA2 v.1.11.5 bioinformatic software package to ascertain the number of unique amplicon sequencing variants (ASV) contained in each sample [62]. This workflow was adapted from the DADA2 analysis described at www.nemabiome.ca, with modifications to accommodate the β-tubulin amplicons analyzed in this paper. Briefly, FASTQ files were prefiltered with the ‘FilterAndTrim’ function to remove any ‘N’s contained in the sequences. Cutadapt was used to remove forward and reverse primer sequences in the amplicons [63]. After primer removal, reads were filtered again with ‘FilterAndTrim’ with no N’s allowed, maxEE = 6, truncQ = 2, a minimum length of 200 bp for each forward and reverse read, and the removal of phiX if identified. As the *Alu/Asu-bt-B* amplicon is >600 bp, the paired-end reads do not have any overlap; resultantly the reads were trimmed to a length of 278 bp, to ensure all amplicons are merged at a consistent length. The error profile of the reads was assessed and reads denoised accordingly using ‘learnErrors’ and ‘derepFastq’ respectively. Overlapping reads were merged with the mergePairs function, while amplicons without paired read overlap (i.e *Alu/Asu-bt-B*) were merged with ‘justConcatenate = TRUE’ and ‘NNNNNNNNNN’ placed between the paired reads to denote the forced merger. A sequence table was constructed with ‘makeSequenceTable’ to display all ASVs present in the dataset. Chimeras were removed with ‘removeBimeraDenovo’. This provides a read count of each ASV present in each sample. A fasta file was also generated with the ‘getUniques’ and ‘uniquesToFasta’ commands to provide a list of all ASVs identified and their corresponding nucleotide sequence. Each ASV was blasted against a reference sequence to ensure that the ASV correctly matched the intended amplicon. Any off-target amplicons were subsequently removed from analysis. Furthermore, 21 ASVs with extremely low reads (below 40) and only detected in one or two worms were manually deleted from the *Alu/Asu-bt-B* dataset since these were most likely PCR artifacts. The resulting sequence list was then screened for the presence of any canonical resistance conferring single nucleotide polymorphisms (SNPs).

Worm genotypes were defined by applying a read count threshold of 1,000 reads. ASVs below threshold level were excluded from further population genetic analysis. As amplicons were generated from single adult worms, a maximum of two ASVs was expected from each sample. There was one *A*. *lumbricoides* worm with only reads below threshold and five *A*. *suum* worms with reads above threshold for more than two ASVs, these six worms were not included in the genotype data used. Basic population genetic analysis was conducted in R Studio (v1.3.1093) [64] using the package PopGenReport [65,66]. Allele counts and allele richness by locus and by population were calculated. Likewise, pairwise FSTs were estimated according to Nei [67].

## Results

### Characterization and comparison of the β-tubulin gene families

Seven β-tubulin genes and an eighth putative β-tubulin encoding gene were identified for both *A*. *lumbricoides* and *A*. *suum*. **Table 2** gives an overview of the proposed and previously used nomenclature for the different genes of both species. Regarding the nomenclature used in this analysis; for *A*. *lumbricoides* genes (PRJEB4950) the prefix ‘Alu’ is used, while *A*. *suum* genes (PRJNA62057) start with ‘Asu’. β-tubulin is abbreviated by ‘bt’. Genes closely related in both species are named correspondingly (e.g. *Alu-bt-A* and *Asu-bt-A*). The use of Latin alphabet characters over numbers was preferred as the use of numbers as indexes may lead to erroneous assumptions regarding orthology with genes of other species. For example *Cel-tbb-1* and *Hco-tbb-iso-1* are not orthologous.

**Table 2:** Alignment of the proposed with the previously used nomenclature for β-tubulins of *Ascaris* species.

Accession number	Nomenclature used in this analysis	Previous nomenclature	References
***Ascaris lumbricoides***			
ALUE_0000927201	*Alu-bt-A*	β-tubulin	Diawara et al., 2009 [41] Diawara et al., 2013a [35] Diawara et al., 2013b [36] Zuccherato et al., 2018 [39] Matamoros et al., 2019 [40]
		*Alutbb-1*	Demeler et al., 2013 [15]
		*tbb-1*.*2*	Krücken et al., 2017 [34]
		β-tubulin isotype 1	Rashwan et al., 2017 [37] Furtado et al., 2019 [38]
ALUE_0000986501	*Alu-bt-B*	*tbb-2*	Krücken et al., 2017 [34]
ALUE_0001827701	*Alu-bt-B’*	*-*	-
ALUE_0000494801	*Alu-bt-C*	*tbb-1*	Krücken et al., 2017 [34]
ALUE_0001031701	*Alu-bt-D*	*-*	-
ALUE_0000949301	*Alu-bt-E*	*tbb-4*	Krücken et al., 2017 [34]
ALUE_0000949201	*Alu-bt-F*	*-*	-
ALUE_0001294101	*Alu-bt-G*	*-*	-
***Ascaris suum***			
GS_23993	*Asu-bt-A*	*tbb-1*.*2*	Krücken et al., 2017 [34]
		β-tubulin	Palma et al., 2020 [42]
GS_01240	*Asu-bt-B*	*Asutbb-2 (Asutbb-3)*	Demeler et al., 2013 [15]
- *	*Asu-bt-B’* *	*-*	-
GS_11145	*Asu-bt-C*	*Asutbb-1*	Demeler et al., 2013 [15]
GS_13691	*Asu-bt-D*	*-*	-
GS_05353	*Asu-bt-E*	*Asutbb-4*	Demeler et al., 2013 [15]
GS_11773	*Asu-bt-F*	*-*	-
GS_10401 & GS_22804	*Asu-bt-G*	*-*	-

For *Ascaris lumbricoides* genes (PRJEB4950) the prefix ‘Alu’ is used, while *Ascaris suum* genes (PRJNA62057) start with ‘Asu’. β-tubulin is abbreviated by ‘bt’. Genes closely related in both species are named correspondingly. *: The amplicon sequencing results from this study suggested that *bt-B’* is present in both *A*. *lumbricoides* and *A*. *suum*, even though it was only identified in the former species in the public datasets. Further research is needed to clarify if *Alu/Asu-bt-B’* should be considered as a separate β-tubulin gene or as an allelic variant of *Alu/Asu-bt-B*.

**Fig 2:**
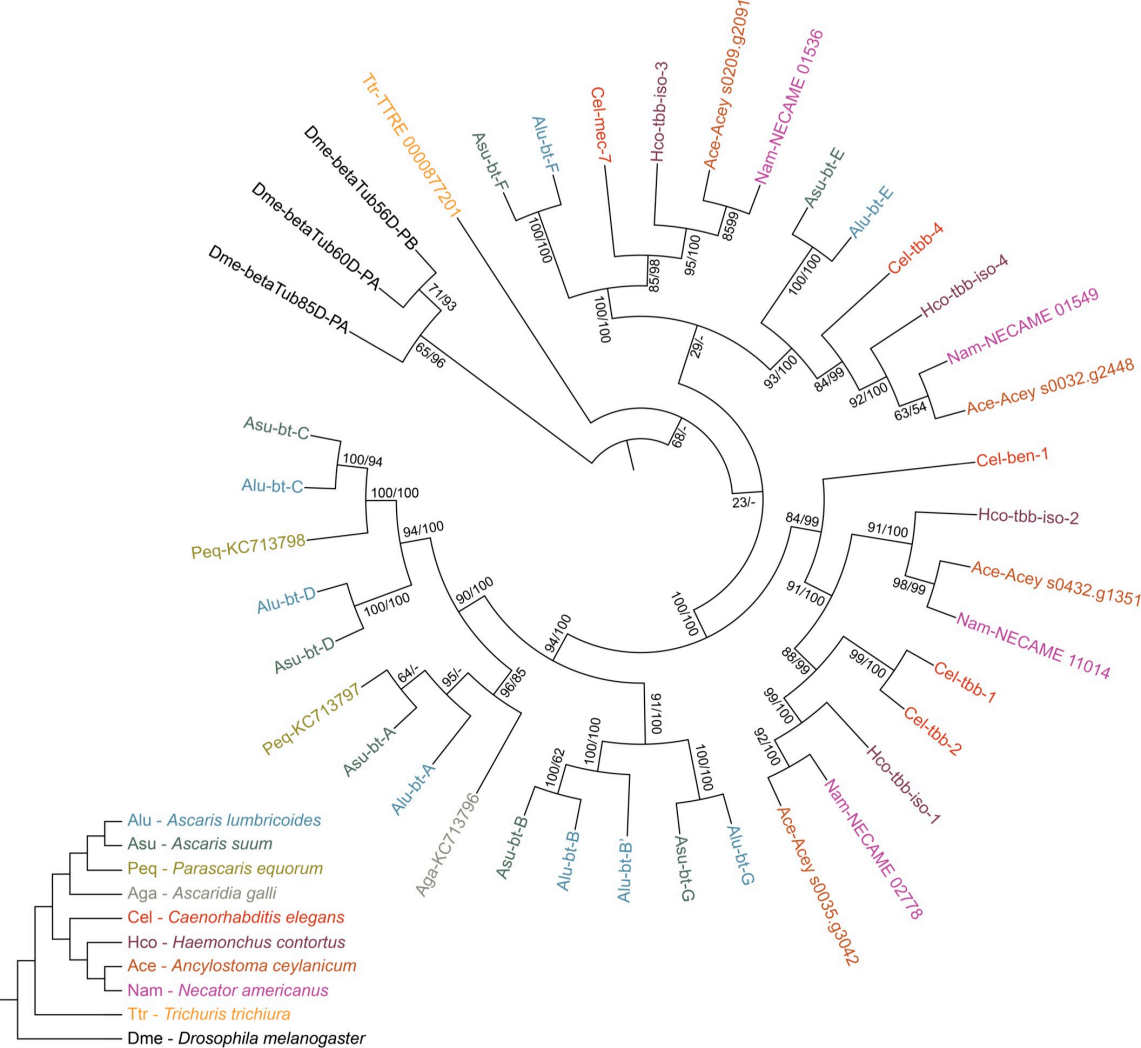
Phylogenetic tree of β-tubulin proteins of *Ascaris* species, other parasitic nematodes, and *C*. *elegans*. Phylogenies were reconstructed with RAxML and MRBAYES. The topology shown is from RaxML. The node support values are percent bootstraps / posterior probabilities. The ‘-‘ indicates that a node was not present in the MRBAYES tree. The branches are equal length to show topology clearly; a tree with branch lengths showing divergence is available in supplementary materials (S14 Info).

Phylogenetic analysis based on amino acid sequences (**Fig 2**) showed that the β-tubulin gene family is highly conserved in the two species (β-tubulin (bt) A, B, C, D, E, F and G). Additionally, an eighth putative β-tubulin encoding gene was observed in the published genome for *A*. *lumbricoides* but was absent in *A*. *suum*. However, due to its close relationship with *Alu-bt-B* it was not clear if this sequence had to be considered as an extra gene or as an allelic variant of *Alu-bt-B*. The sequence was therefore named *Alu-bt-B’*. Furthermore, the amplicon sequencing results from this study contained an ASV of *Alu/Asu-bt-B* (btB-ASV26) also generated from *A*. *suum* samples which showed only one nucleotide difference with *Alu-bt-B’*. The results therefore suggest that *Alu/Asu-bt-B’* is present in both *A*. *lumbricoides* and *A*. *suum*. Alignment of complete amino acid sequences of the β-tubulins from the published genomes is provided in **S5 Info**.

To understand the evolutionary relationship of β-tubulins from both human and animal STHs, a protein alignment was generated, including *A*. *suum*, *A*. *lumbricoides*, *P*. *equorum* and *A*. *galli* (belonging to Clade III in the nematode phylogeny); *T*. *trichiura* (Clade I); *H*. *contortus*, *A*. *ceylanicum* and *N*. *americanus* (Clade V), and the free-living *C*. *elegans* (Clade V) (**S1 Info**). Both maximum likelihood and Bayesian phylogenetic trees returned similar topologies that closely followed the species phylogeny (**Fig 2**). *A*. *lumbricoides* and *A*. *suum* have, similar to *H*. *contortus*, *A*. *ceylanicum* and *N*. *americanus*, genes that are orthologues to *C*. *elegans mec-7* and *tbb-4 (Alu/Asu-bt-E* and *bt-F)*. The relationship to the other *C*. *elegans* genes is more complex. *H*. *contortus*, *A*. *ceylanicum* and *N*. *americanus* have two sets of clear one-to-one orthologues, but inclusion of *C*. *elegans* suggests a pattern of gene duplication and loss. Resolution of these relationships requires analyses with more species, which are currently being performed. The *Ascaris* species show a lineage specific expansion, with at least six gene duplication events, one each for *Alu/Asu-bt-A*, *bt-B*, *bt-C*, *bt-D*, *bt-G* and then another giving *Alu-bt-B’*. The included β-tubulins of *P*. *equorum* and *A*. *galli*, both members of Clade III, show a close phylogenetic relationship with the genes of *Ascaris*. It has to be noted that for these two species only the sequences published by Tyden et al. [26] were included and no further genomic or transcriptomic data was investigated for other potential β-tubulin genes orthologues to *C*. *elegans mec-7* and *tbb-4*.

**Table 3** gives an overview of the three codons of particular interest for the detection of BZ resistance associated SNPs (167, 198 and 200). BZ susceptible genotypes were present at codons 167 and 198 in all the *A*. *lumbricoides* and *A*. *suum* β-tubulin gene family members; Phe (TTC) at codon 167 and Glu (GAA or GAG) at codon 198. In contrast, at codon 200, the BZ susceptible genotypes Phe (TTC or TTT) were present in all the genes except for *Alu/Asu-bt-D*, which has the BZ resistance genotype Tyr (TAT).

**Table 3:** Genotype of codons 167, 198 and 200 in the identified *Ascaris* β-tubulin genes.

Codon	Genotype	Amino acid	*Ascaris* β-tubulin genes
**167**	TTC	Phe	All identified *Ascaris* β-tubulin genes
**198**	GAA	Glu	*Alu-bt-A* and *Asu-bt-A* *Alu-bt-B* and *Asu-bt-B* *Alu-bt-B’* and Asu-bt-B’* Alu-bt-C *Alu-bt-D* and *Asu-bt-D* *Alu-bt-F* and *Asu-bt-F* *Alu-bt-G* and *Asu-bt-G*
	GAG	Glu	*Asu-bt-C* *Alu-bt-E* and *Asu-bt-E*
**200**	TTC	Phe	*Alu-bt-A* and *Asu-bt-A* *Alu-bt-B* and *Asu-bt-B* ** Alu-bt-B’ and Asu-bt-B’* Alu-bt-C *Alu-bt-E* and *Asu-bt-E* *Alu-bt-F* and *Asu-bt-F* *Alu-bt-G* and *Asu-bt-G*
	TTT	Phe	*Alu-bt-B* and *Asu-bt-B* ** Asu-bt-C
	TAT	Tyr	*Alu-bt-D* and *Asu-bt-D*

*: *Asu-bt-B’* was sequenced from *A*. *suum* worms in this study and showed the same genotypes as *Alu-bt-B’*. **: A synonymous sequence polymorphism was observed at codon 200 of *Alu/Asu-bt-B* in both *A*. *lumbricoides* and *A*. *suum* sequenced in this study (btB-ASV01, btB-ASV03 and btB-ASV13). This polymorphism was not concluded from the published genomes, in which TTC was present in *Asu-bt-B* and TTT in *Alu-bt-B*.

### Transcription profiles of the β-tubulin genes during the *Ascaris* life cycle

**Fig 3:**
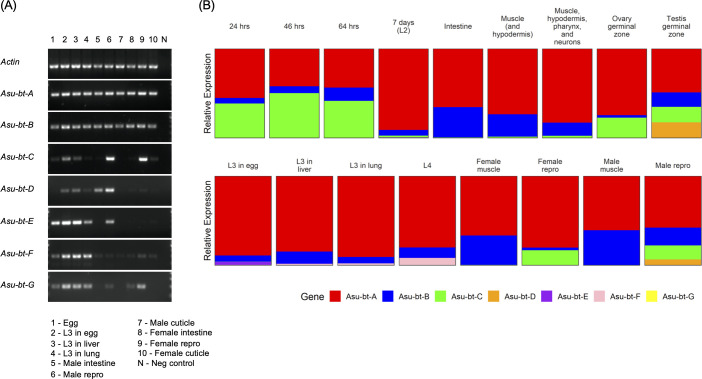
Transcription profiles of the β-tubulins of *Ascaris suum*. **Panel A.** Reverse transcription (RT)-PCR analyses were performed on cDNA samples with gene specific primer sets. The agarose gels show the transcription profiles of the β-tubulins in four developmental stages (egg, third larval stage (L3) in egg, L3 in liver, L3 in lung) and three tissue types of adult male and female worms (intestinal, reproductive, and cuticle). **Panel B.** RNA-seq analyses were performed using RNA-seq data obtained from the NCBI Sequence Read Archive. The stacked bar charts show the expression of each β-tubulin (relative to total β-tubulin expression) in different developmental stages (24h embryos to L4 stage larvae) and different tissue types of *A*. *suum*.

RT-PCR was performed for each of the seven identified β-tubulin genes for multiple life cycle stages and tissues of *A*. *suum* (**Fig 3**, panel A) and the expression of these genes was also examined from the available RNA-seq data (**Fig 3**, panel B). Both the analyses indicated that only two of the seven identified β-tubulin genes are highly and widely expressed. The gene *Asu-bt-A* amplified by RT-PCR from cDNA from all life stages and tissues of the parasite and had consistently high RNA-seq expression levels. Also the gene *Asu-bt-B* is expressed throughout the entire life cycle of the parasite albeit at lower levels than *Asu-bt-A*. All other β-tubulin genes were transcribed in specific life stages or tissues, suggesting more specialized functions (**Fig 3**). For some genes, although RT-PCR amplification confirmed the expression in certain tissues, the RNA-seq data clearly indicated low transcription levels. Based on these results, *Alu/Asu-bt-A* and *Alu/Asu-bt-B* were selected as the most interesting targets for BZ mode of action and subsequently the most relevant for screening for potential resistance mutations. Consequently, these two genes were selected for deep amplicon sequencing to assess intra- and inter-species genetic diversity and the presence of sequence polymorphisms at codons 167, 198 and 200.

### Intra- and inter-species genetic diversity and presence of candidate BZ resistance associated SNPs in drug-exposed *A*. *lumbricoides* and *A*. *suum* populations

**Fig 4:**
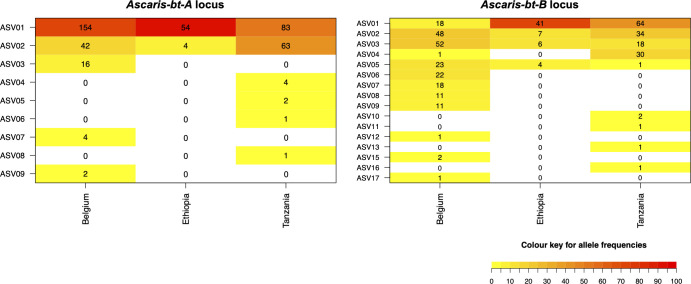
Allele counts for *Alu/Asu-bt-A* and *Alu/Asu-bt-B* in three adult *Ascaris* worm populations. *Ascaris suum* worms were collected from the intestines of pigs slaughtered in Belgium. *Ascaris lumbricoides* worms were collected during two expulsion studies in Ethiopia and Tanzania. The total number of each amplicon sequence variant (ASV) per population is displayed (allele count) and the color indicates the proportion within the population (allele frequency). Heatmaps were created using the R package PopGenReport [65,66].

Deep amplicon sequencing was successfully performed on *Alu/Asu-bt-A* and *Alu/Asu-bt-B* for 215 and 209 adult *Ascaris* worms respectively, applying a read threshold of 1,000 reads. The amplicon sequencing data for *Alu/Asu-bt-A* clearly suggested the primers used target a single locus since there was a maximum of two β-tubulin ASVs amplified from each single worm (**S6 Info**). The data from *Alu/Asu-bt-B* was somewhat more complex. While the majority of single worms had a maximum of two ASVs with high read counts (consistent with a diploid genotype), 47.7% (52/109) of *A*. *suum* worms and 15.1% (16/106) of *A*. *lumbricoides* worms had several additional β-tubulin ASVs at very low read depths (**S11 Info**). Although additional low frequency ASVs can be generated from samples due to bar-code hopping, there was no pattern related to particular index combinations and so it is more likely to be due to “off target” amplification from a second copy of *Alu/Asu-bt-B* and other β-tubulin genes. For example, ASVs corresponding to the *Alu/Asu-bt-A* locus (btA-ASV01, btA-ASV02, btA-ASV08) were also detected in the *Alu/Asu-bt-B* Illumina samples, but only two worms showed read counts above threshold level (1,000 reads). Both loci appeared to be relatively conserved, with *Alu/Asu-bt-A* being less diverse than *Alu/Asu-bt-B*. Allelic richness by locus and population is displayed in **Table 4**. Overall, the Tanzanian and Belgian *Ascaris* populations show more diversity than the Ethiopian population. The intra- and inter-population genetic diversity of both loci is graphically presented in **Fig 4** as heatmaps.

**Table 4:** Allelic richness of *Alu/Asu-bt-A* and *Alu/Asu-bt-B* in the investigated human and porcine adult *Ascaris* worm populations.

Allelic richness	*Ascaris suum*	*Ascaris lumbricoides*	
	(n = 109)	Ethiopia (n = 29)	Tanzania (n = 77)
*Alu/Asu-bt-A*	4.112	1.997	4.067
*Alu/Asu-bt-B*	9.156	3.997	5.956
Mean	6.634	2.997	5.011
total	13.268	5.994	10.022

Allelic richness for both *Alu/Asu-bt-*A and *Alu/Asu-bt-B* in each of the three populations (*Ascaris suum* in Belgium and *Ascaris lumbricoides* in Ethiopia and Tanzania). Results were calculated using the R package PopGenReport [65,66].

For the *Alu/Asu-bt-A* locus, only nine ASVs were identified across the three populations (**S6 Info**). These were highly similar with a percentage of identity being > 99% for all pair-wise comparisons (**S7 Info**). All variation in the sequenced region was located within the intron. No F167Y (TTC>TAC or TTT>TAT), E198A (GAA>GCA or GAG>GCG), E198L (GAA>TTA) and F200Y (TTC>TAC or TTT>TAT) BZ resistance associated SNPs were found in any of the ASVs (**S8 Info**). Two ASVs (btA-ASV01 and btA-ASV02) were highly frequent in all three *Ascaris* populations with a total coverage of 93.0% (400/430) of all sequenced fragments (**S9 Info**). The most common worm genotypes were the same within the human and porcine *Ascaris* populations.

For the *Alu/Asu-bt-B* locus, a total of 21 ASVs were detected in the complete dataset, of which only 16 were included in the population genetic analysis based on the minimum threshold of 1,000 reads (**S11 Info**). Among the 21 ASVs, the percentage identity ranged from 94.6% to 98.5% and nucleotide variation in this fragment was located in both intron and exon (**S12 Info** and **S13 Info**). Two ASVs (btB-ASV23 and btB-ASV26) having only low read counts (< 1,000) in a total of seven worms contained SNPs resulting in amino-acid substitutions (P173H (CCT>CAT), Q191E (CAG>GAG), V193I (GTT>ATT) and D197N (GAT>AAT)). All other polymorphisms were synonymous. Considering the SNPs that could be potentially associated with BZ resistance, as for *Alu/Asu-bt-A*, no F167Y (TTC>TAC or TTT>TAT), E198A (GAA>GCA or GAG>GCG), E198L (GAA>TTA) or F200Y (TTC>TAC or TTT>TAT) mutations were detected. Although, a synonymous sequence polymorphism was present at codon 200 in three ASVs (btB-ASV01, 03 and 13) having the Phe (TTT) instead of the Phe (TTC) genotype (**S13 Info**). More detailed data regarding genotype counts and frequencies can be found in supplementary materials (**S10 Info**).

Based on the two β-tubulin loci, Nei’s pairwise FST values between all pairs of populations were calculated; Belgium-Ethiopia: 0.0710, Belgium-Tanzania: 0.0532, Ethiopia-Tanzania: 0.0713 [67]. These results reveal low levels of population differentiation and suggest that among the investigated populations, the *A*. *suum* population collected from pigs is not the most divergent.

## Discussion

### At least seven β-tubulin genes were identified for both *A*. *lumbricoides* and *A*. *suum*

This study demonstrated the presence of at least seven β-tubulin genes in *Ascaris* species. All the identified β-tubulin gene family members, except *Alu/Asu-bt-D*, have the susceptible genotype at codons 167, 198 and 200. Of the possible candidates, most do not have one-to-one orthologous relationships with *C*. *elegans* and *H*. *contortus* β-tubulin genes. Thus, prioritizing the genes most likely to play a potential role in BZ resistance by simple extrapolation of information from those species is difficult. The only two exceptions to this are *Alu/Asu-bt-E* and *bt-F* which appear orthologous to *Cel-tbb-4* and *Cel-mec-7* respectively. These *C*. *elegans* genes have very specialized functions and are only expressed in a small subset of cells including specific sensory neurons and do not appear to be involved in BZ resistance [68,69]. Consistent with this, *Alu/Asu-bt-E* and *bt-F* are only expressed at very low levels and so it seems reasonable to assume these are not likely to be involved in BZ resistance. In the phylogenetic analysis, we noted high rates of variation in the three pairs *Alu/Asu-bt-C*, *bt-D*, and *bt-G*, which is indicative that these genes are under different selective pressures from *Alu/Asu-bt-A* and *bt-B*. It might suggest that these three more rapidly evolving genes are adapting to a specific cell or tissue in *Ascaris*, analogous to *Cel-mec-7* and *Cel-tbb-4* in *C*. *elegans*, and *Hco-tbb-iso-3* in *H*. *contortus* [27,68,70]. Only two of the β-tubulin genes, namely *Alu/Asu-bt-A* and *Alu/Asu-bt-B*, are widely expressed in all stages of the parasite’s lifecycle and all tissues of adult worms. Consequently, we considered these the most likely relevant BZ targets.

Regarding the place of *Alu/Asu-bt-B’* in the *Ascaris* β-tubulin gene family, the results of the deep amplicon sequencing of *Alu/Asu-bt-B* showed two ASVs almost identical to the *Alu-bt-B’* reference sequence (btB-ASV23 and btB-ASV26). These ASVs only appeared at low read counts in nine *w*orms in addition to two highly frequent *Alu/Asu-bt-B* ASVs. Four SNPs present in *Alu-bt-B’*, btB-ASV23 and btB-ASV26 result in amino-acid substitutions (P173H (CCT>CAT), Q191E (CAG>GAG), V193I (GTT>ATT) and D197N (GAT>AAT)). Further research is needed to clarify if this sequence should be considered as an allelic variant of *Alu/Asu-bt-B* or as a separate β-tubulin gene, although the results of the present study suggest the latter since both observations would be unusual for alleles of the same gene.

### There is a need for a new nomenclature for β-tubulins in nematodes

Similar to previous studies, the presented data suggested that diversification of β-tubulins occurred independently in different nematode lineages. Hence, β-tubulin families are more conserved between species of the same nematode clade. For *Ascaris* species in particular, this complex phylogeny has the consequence that the currently used nomenclature is confusing. More specifically, the term *Ascaris β-tubulin isotype 1*, used for the most frequently studied gene, falsely suggests homology with *Hco-tbb-iso-1* of *H*. *contortus* or *Cel-tbb-1* of *C*. *elegans*. To avoid such incorrect assumptions regarding phylogenetic relationships and potential orthology, we have used letters as indexes to name the β-tubulins identified in the present study. In relation to this, a comprehensive nomenclature of β-tubulin genes in parasitic nematodes that considers the complex phylogeny and the continuous expansion of knowledge is needed.

### *Alu/Asu-bt-A* has less allelic diversity than *Alu/Asu-bt-B*

The genetic diversity of the *Alu/Asu-bt-A* and *bt-B* genes was investigated in adult worms collected from porcine and human populations using deep amplicon sequencing. The sequence diversity in the *Alu/Asu-bt-A* locus within and between worm populations was markedly lower than for *Alu/Asu-bt-B*. For *Alu/Asu-bt-A* one haplotype (btA-ASV01) was by far the most frequent in all three populations (adult *A*. *suum* worms from Belgium and adult *A*. *lumbricoides* worms from Ethiopia and Tanzania). This is consistent with the very close phylogenetic relationship between *A*. *lumbricoides* and *A*. suum, and probably represents ancestral polymorphism. The higher diversity in the porcine population for both *Alu/Asu-bt-A* and *bt-B* may be explained by the fact that *A*. *suum* worms were randomly collected from the intestines of pigs slaughtered in different slaughterhouses in Belgium. Although there is no information about the farms where the pigs were raised, a diverse origin and a broad distribution among a number of herds is assumed, each with a different management and treatment history. In contrast, human *Ascaris* worms were collected only from two schools. The reason for the higher genetic diversity of both *Alu/Asu-bt-A* and *bt-B* loci in the Tanzanian versus the Ethiopian population is unclear and might suggest a larger effective population size of the parasite in Tanzania, e.g. due to higher infection intensities [71]. Yet, it is also possible that the observed difference is a consequence of the smaller worm population size, sampled from a smaller number of children in the Ethiopian school.

### Candidate BZ resistance SNPs are absent in *Alu/Asu-bt-A* and *Alu/Asu-bt-B* in adult *A*. *lumbricoides* and *A*. *suum* worms

All the individual adult *A*. *lumbricoides* worms from Ethiopia and Tanzania that were sequenced had the BZ susceptible genotype at codons 167, 198 and 200 since the F167Y (TTC>TAC or TTT>TAT), E198A (GAA>GCA or GAG>GCG), E198L (GAA>TTA) and F200Y (TTC>TAC or TTT>TAT) polymorphisms were not observed in either of the two targeted genes. Diawara and colleagues were previously able to identify the mutation F167Y (TTC>TAC) in the gene *Alu/Asu-bt-A* in *A*. *lumbricoides* worm eggs collected both before and after treatment in Haiti, Kenya and Panama [35]. Furtado and colleagues were the first to report the mutation TTC>TAC at codon 200 of *Alu/Asu-bt-A* in *A*. *lumbricoides* eggs collected in seven Brazilian states, but only at a very low frequency of 0.5% (4/854) [38]. For the set of *A*. *lumbricoides* worms investigated in this study, the presence of the potentially BZ resistance associated SNPs was not expected since the worms were collected after expulsion by treatment with BZ drugs and so were susceptible to the drug. Based on the reported national coverage of drug administration for the last 5 years, the site in Ethiopia was considered to have experienced a low drug pressure with MDA administered to SAC since 2015 [72]. The school in Tanzania has a history of MDA since 1994 and was therefore considered as high drug pressure region [70]. Recently, Vlaminck et al. reported an efficacy of ALB against *A*. *lumbricoides* of 99% in Ethiopia and 96.8% in Tanzania [71].

Similarly, the F167Y (TTC>TAC or TTT>TAT), E198A (GAA>GCA or GAG>GCG), E198L (GAA>TTA) and F200Y (TTC>TAC or TTT>TAT) polymorphisms were not observed in either of the two targeted genes in any of the *A*. *suum* adult worms sequenced from Belgium. Regarding *A*. *suum*, there are no reports from the pig sector describing declined efficacy of BZs to date, even though the drugs have been widely used for decades to control infections. However, the fact that *Ascaris* has minimal acute clinical signs, may result in a lack of recognition of treatment failure in the field. The result of 100% wild-type alleles is in agreement with the study of Palma et al. (2020) likewise unable to demonstrate the presence of SNPs associated with BZ resistance in *A*. *suum* collected from pigs [42].

### Conclusion

Accurate and reliable detection of molecular markers of BZ resistance in STHs will be critical in the upcoming years, anticipating the continuing increase in number of drug treatments to reach the WHO target in all STH-endemic countries. The characterization of the β-tubulin family in *Ascaris* species provides a framework to investigate the prevalence and potential role of β-tubulin sequence polymorphisms in BZ resistance in a more systematic manner than previously possible. The work has revealed that *Alu/Asu-bt-A* and *Alu/Asu-bt-B* are the obvious β-tubulin genes to prioritize in this context. Nevertheless, further research into the associations between the frequency of SNPs, the drug efficacy assessed by egg counts and the history of drug pressure on investigated worm populations will allow substantiation of the role of the different β-tubulin gene family members in BZ resistance and validation of particular SNPs as molecular markers for BZ resistance.

## Supporting information

S1 Info β-tubulin proteins included in phylogenetic analyses. WormBase ParaSite or NCBI Gene ID and Transcript ID of all β-tubulin proteins included in the phylogenetic analyses and trees. *Caenorhabditis elegans* and *Haemonchus contortus* β-tubulin protein sequences were obtained from published genomes [50,73]. The corresponding names were adopted from published literature [27,30]. For *Trichuris trichiura*, *Necator americanus* and *Ancylostoma ceylanicum* keyword search for ‘tubulin beta’ and BLASTP search with the *Ascaris* β-tubulin genes against the published genomes [74–76] resulted in two sequences for *T*. *trichiura* and four sequences for both hookworms. The genome assembly and annotation of *A*. *ceylanicum* were used as close representative of *A*. *duodenale*, since this genome assembly has low quality metrics. For *Parascaris equorum* and *Ascaridia galli*, the sequences published by Tyden et al. (2013) were used [26]. Three β-tubulins from *Drosophila melanogaster* are included in the list to be used as outgroups.
(XLSX)

S2 Info Reverse transcription (RT)-PCR Primers. β-tubulin gene specific primers used in RT-PCR analysis. All β-tubulin primers are based on coding sequences. The actin gene is used as household gene and primers are adopted from Vlaminck et al., 2011 [56].
(PDF)

S3 Info *Ascaris* RNA-seq datasets used. Overview of all RNA-seq datasets (NCBI Sequence Read Archive (SRA)) used in the *A*. *suum* β-tubulin RNA-seq analysis.
(XLSX)

S4 Info Illumina adapted primers for *Alu/Asu-bt-A* and *Alu/Asu-bt-B* (and *bt-B’*) and Illumina primers with barcode indices and P5/P7 sequencing regions. *Alu/Asu-bt-A* and *Alu/Asu-bt-B* (and *bt-B’*) primers with Illumina Adapters. Locus specific primer sequence bolded, N’s underlined. Illumina adaptor oligonucleotide sequences were obtained from the Illumina Adapter Sequences document dated March 2020 (Illumina Inc.). Forward and Reverse barcoded sequencing primers. Index sequence bolded. Sequences were obtained from the Illumina Adapter Sequences document dated March 2020 (Illumina Inc.).
(PDF)

S5 Info *Ascaris* β-tubulin protein alignment. Alignment of amino acid sequences of the identified β-tubulins of both *A*. *suum* and *A*. *lumbricoides*. For aesthetic reasons, all sequences were trimmed to a uniform length of 427 amino acids. (N-terminus trimmed: *Asu-bt-B*, *Asu-bt-E*; C-terminus trimmed: all).
(PDF)

S6 Info Amplicon sequencing results for *Alu/Asu-bt-A*. Excel spreadsheet of amplicon sequencing results for *Alu/Asu-bt-A* following analysis with the DADA2 v.1.11.5 bioinformatic software package and manual assessment for chimeras, off-target amplicons and PCR artefacts. The first column of the spreadsheet contains the single worm identification. In the adjacent columns the read number for each unique amplicon sequence variant (ASV) is given. Corresponding genotypes were defined by applying a threshold of 1,000 reads. ASVs below threshold level were excluded from further population genetic analyses and are indicated in red in the table. There were no worms with reads above the threshold for more than two ASVs.
(XLSX)

S7 Info Summary of pairwise distances assessment of nine *Alu/Asu-bt-A* ASVs. Percentage of bases/residues which are identical (% Identity), and number of bases/residues which are identical (# Identities) and not identical (# Differences). Results for the nine identified amplicon sequence variants (ASVs) of *Alu/Asu-bt-A*. All pairwise distances were computed using Geneious v10.2.6.
(XLSX)

S8 Info Nucleotide alignment of *Alu/Asu-bt-A* ASVs. Nucleotide alignment of the nine identified amplicon sequence variants (ASVs) of *Alu/Asu-bt-A*. Multiple alignment was performed using Geneious v10.2.6. The three codons of interest in the context of benzimidazole resistance are indicated by their number at the base of the consensus sequence.
(PDF)

S9 Info Allele counts and frequencies of both *Alu/Asu-bt-A* and *Alu/Asu-bt-B*. The total number of each amplicon sequence variant (ASV) (allele count) and the proportion within the (sub)populations (allele frequency) for both *Alu/Asu-bt-A* and *Alu/Asu-bt-B*. *A*. *suum* worms were collected from the intestines of pigs slaughtered in Belgium. *A*. *lumbricoides* worms were collected during two expulsion studies in Ethiopia and Tanzania.
(XLSX)

S10 Info Genotype counts and frequencies of both *Alu/Asu-bt-A* and *Alu/Asu-bt-B*. The total number of each defined genotype (genotype count) and the proportion within the (sub)populations (genotype frequency) for both *Alu/Asu-bt-A* and *Alu/Asu-bt-B*. Genotype per worm was defined by applying a threshold of 1,000 reads. *A*. *suum* worms were collected from the intestines of pigs slaughtered in Belgium. *A*. *lumbricoides* worms were collected during two expulsion studies in Ethiopia and Tanzania.
(XLSX)

S11 Info Amplicon sequencing results for *Alu/Asu-bt-B*. Excel spreadsheet of amplicon sequencing results for *Alu/Asu-bt-B* following analysis with the DADA2 v.1.11.5 bioinformatic software package and manual assessment for chimeras, off-target amplicons and PCR artefacts. The first column of the spreadsheet contains the single worm identification. In the adjacent columns the read number for each unique amplicon sequence variant (ASV) is given. From the dataset, 21 ASVs with extremely low reads (below 40) and only detected in 1 or 2 worms were manually deleted since these were seen as PCR artefacts. Furthermore, due to a lack of complete specificity of the primers, the dataset contained 10 ASVs corresponding to the *Alu/Asu-bt-A* locus, which were also manually deleted. The genotype per worm was defined by applying a threshold of 1,000 reads. ASVs below threshold level were excluded from further population genetic analyses and are indicated in red in the table. There was one *A*. *lumbricoides* worm with only reads below threshold and five *A*. *suum* worms with reads above threshold for more than two ASVs, these six worms were not included in the genotype data used for population genetic analysis.
(XLSX)

S12 Info Summary of pairwise distances assessment of 21 *Alu/Asu-bt-B* ASVs. Percentage of bases/residues which are identical (% Identity), and number of bases/residues which are identical (# Identities) and not identical (# Differences). Results for the 21 identified amplicon sequence variants (ASVs) of *Alu/Asu-bt-B*. All pairwise distances were computed using Geneious v10.2.6.
(XLSX)

S13 Info Nucleotide alignment of *Alu/Asu-bt-B* ASVs. Nucleotide alignment of the 21 identified amplicon sequence variants (ASVs) of *Alu/Asu*-bt-B. Multiple alignment was performed using Geneious v10.2.6. The three codons of interest in the context of benzimidazole resistance are indicated by their number at the base of the consensus sequence.
(PDF)

S14 Info Phylogenetic tree of β-tubulin proteins of *Ascaris* species, other parasitic nematodes and *C*. *elegans*. Phylogenies were reconstructed with RAxML and MRBAYES. The topology shown is from RAxML. The node support values are percent bootstraps / posterior probabilities. The ‘-‘ indicates that a node was not present in the MRBAYES tree. The branch lengths show divergence.
(PDF)
