# Supplementary material for: Characterization of the β-tubulin gene family in Ascaris lumbricoides and Ascaris suum and its implication for the molecular detection of benzimidazole resistance
Source: PLoS Negl Trop Dis. 2021 Sep 27;15(9):e0009777. doi: 10.1371/journal.pntd.0009777 (PMC8496844; doi:10.1371/journal.pntd.0009777)
Supplement: S2 Info — β-tubulin gene specific primers used in RT-PCR analysis. All β-tubulin primers are based on coding sequences. The actin gene is used as household gene and primers are adopted from Vlaminck et al., 2011 [56]. (PDF) [file pntd.0009777.s002.pdf]

S2 Info

β-Tubulin gene specific primers used in RT-PCR analysis. All β-tubulin primers are based on coding sequences. The actin gene is used as household gene and primers adopted from Vlaminck et al., 2011 [1].

Primer ID	Gene nomenclature used in this study	Gene accession WormBase ParaSite or Reference	Primer Sequence	nmol	Tm (°C)
Asu_GS11145_for	*Asu-bt-C*	GS_11145	GCCTACGGAGATCTCAACCA	28	56.3
Asu_GS11145_rev	*Asu-bt-C*	GS_11145	ATATCGCCGCCACAGTAAGA	28.1	56
Asu_GS01240_for	*Asu-bt-B*	GS_01240	GGACAATGCGGCAATCAGAT	32.7	55.9
Asu_GS01240_rev	*Asu-bt-B*	GS_01240	TGACCCTTCGCCCAGTTATT	35.4	56.4
Asu_GS23993_for	*Asu-bt-A*	GS_23993	TTGGAGCCTTACAACGCAAC	25	55.9
Asu_GS23993_rev	*Asu-bt-A*	GS_23993	GCGAAACCTGGCATGAAGAA	26.2	56
Asu_GS11773_for	*Asu-bt-F*	GS_11773	CACATACAGGCGGGTCAATG	24.1	56.1
Asu_GS11773_rev	*Asu-bt-F*	GS_11773	TTGTTACCGGCACCACTTTG	32.3	55.9
Asu_GS05353_for	*Asu-bt-E*	GS_05353	ACTGCTGGGCTAAAGGTCAT	37.1	56.4
Asu_GS05353_rev	*Asu-bt-E*	GS_05353	CGTTGCATTGTAGGGCTCAA	22	55.8
Asu_GS22804_for	*Asu-bt-G*	GS10401 + GS_22804	CGCGCTTTTCTTCACTGGTA	31.1	55.6
Asu_GS22804_rev	*Asu-bt-G*	GS10401 + GS_22804	TCCGTATCACCAGCTCCTTC	29.5	56.3
Asu_GS13691_for	*Asu-bt-D*	GS_13691	TTTGCTTCCGGACGCTAAAG	26.7	55.7
Asu_GS13691_rev	*Asu-bt-D*	GS_13691	GGCGACATTGAGTGCTTGAA	25.5	56
Asu_Actin_for	Actin - Housekeeping	Vlaminck et al., 2011 (1)	GGGTCGTGACCTCACTGATT		
Asu_Actin_rev	Actin - Housekeeping	Vlaminck et al., 2011 (1)	TTGATCTTCATCGTGCTTGG		

Reference

1. Vlaminck J, Martinez-Valladares M, Dewilde S, Moens L, Tilleman K, Deforce D, et al. Immunizing pigs with Ascaris suum haemoglobin increases the inflammatory response in the liver but fails to induce a protective immunity. Parasite Immunol. 2011;33(4):250-4.
