# Supplementary material for: Characterization of the β-tubulin gene family in Ascaris lumbricoides and Ascaris suum and its implication for the molecular detection of benzimidazole resistance
Source: PLoS Negl Trop Dis. 2021 Sep 27;15(9):e0009777. doi: 10.1371/journal.pntd.0009777 (PMC8496844; doi:10.1371/journal.pntd.0009777)
Supplement: S4 Info — Alu/Asu-bt-A and Alu/Asu-bt-B (and bt-B’) primers with Illumina Adapters. Locus specific primer sequence bolded, N’s underlined. Illumina adaptor oligonucleotide sequences were obtained from the Illumina Adapter Sequences document dated March 2020 (Illumina Inc.). Forward and Reverse barcoded sequencing primers. Index sequence bolded. Sequences were obtained from the Illumina Adapter Sequences document dated March 2020 (Illumina Inc.). (PDF) [file pntd.0009777.s004.pdf]

S4 Info – *Alu/Asu-bt-A* and *Alu/Asu-bt-B* (and *bt-B'*) Primers with Illumina Adapters.

Locus specific primer sequence bolded, N's underlined. Illumina adaptor oligonucleotide sequences were obtained from the Illumina Adapter Sequences document dated March 2020 (Illumina Inc.).

Primer Name	Primer sequence (5'–3')
BtA-For	**GATCTGGCATGGGAACGT**
BtA-Rev	**CCGTATGTGGGATTTGTAAGC**
BtA-For-Adp-0N	TCGTCGGCAGCGTCAGATGTGTATAAGAGACAG**GATCTGGCATGGGAACGT**
BtA-For-Adp-1N	TCGTCGGCAGCGTCAGATGTGTATAAGAGACAGN**GATCTGGCATGGGAACGT**
BtA-For-Adp-2N	TCGTCGGCAGCGTCAGATGTGTATAAGAGACAGNN**GATCTGGCATGGGAACGT**
BtA-For-Adp-3N	TCGTCGGCAGCGTCAGATGTGTATAAGAGACAGNNN**GATCTGGCATGGGAACGT**
BtA-Rev-Adp-0N	GTCTCGTGGGCTCGGAGATGTGTATAAGAGACAG**CCGTATGTGGGATTTGTAAGC**
BtA-Rev-Adp-1N	GTCTCGTGGGCTCGGAGATGTGTATAAGAGACAGN**CCGTATGTGGGATTTGTAAGC**
BtA-Rev-Adp-2N	GTCTCGTGGGCTCGGAGATGTGTATAAGAGACAGNN**CCGTATGTGGGATTTGTAAGC**
BtA-Rev-Adp-3N	GTCTCGTGGGCTCGGAGATGTGTATAAGAGACAGNNN**CCGTATGTGGGATTTGTAAGC**
BtB-For	**CAATTGACGCACTCGTTGG**
BtB-Rev1	**CGTAAAGTGCCTCGTTATCG**
BtB-Rev2	**CATAGAGCGCCTCGTTATCG**
BtB-For-Adp-0N	TCGTCGGCAGCGTCAGATGTGTATAAGAGACAG**CAATTGACGCACTCGTTGG**
BtB-For-Adp-1N	TCGTCGGCAGCGTCAGATGTGTATAAGAGACAGN**CAATTGACGCACTCGTTGG**
BtB-For-Adp-2N	TCGTCGGCAGCGTCAGATGTGTATAAGAGACAGNN**CAATTGACGCACTCGTTGG**
BtB-For-Adp-3N	TCGTCGGCAGCGTCAGATGTGTATAAGAGACAGNNN**CAATTGACGCACTCGTTGG**
BtB-Rev1-Adp-0N	GTCTCGTGGGCTCGGAGATGTGTATAAGAGACAG**CGTAAAGTGCCTCGTTATCG**
BtB-Rev1-Adp-1N	GTCTCGTGGGCTCGGAGATGTGTATAAGAGACAGN**CGTAAAGTGCCTCGTTATCG**
BtB-Rev1-Adp-2N	GTCTCGTGGGCTCGGAGATGTGTATAAGAGACAGNN**CGTAAAGTGCCTCGTTATCG**
BtB-Rev1-Adp-3N	GTCTCGTGGGCTCGGAGATGTGTATAAGAGACAGNNN**CGTAAAGTGCCTCGTTATCG**
BtB-Rev2-Adp-0N	GTCTCGTGGGCTCGGAGATGTGTATAAGAGACAG**CATAGAGCGCCTCGTTATCG**
BtB-Rev2-Adp-1N	GTCTCGTGGGCTCGGAGATGTGTATAAGAGACAGN**CATAGAGCGCCTCGTTATCG**
BtB-Rev2-Adp-2N	GTCTCGTGGGCTCGGAGATGTGTATAAGAGACAGNN**CATAGAGCGCCTCGTTATCG**
BtB-Rev2-Adp-3N	GTCTCGTGGGCTCGGAGATGTGTATAAGAGACAGNNN**CATAGAGCGCCTCGTTATCG**

S4 Info - Forward and Reverse barcoded sequencing primers.

Index sequence bolded. Sequences were obtained from the Illumina Adapter Sequences document dated March 2020 (Illumina Inc.).

Primer Name	Primer sequence (5'–3')
S502 i5	AATGATACGGCGACCACCGAGATCTACAC**CTCTCTAT**TCGTCGGCAGCGTC
S503 i5	AATGATACGGCGACCACCGAGATCTACAC**TATCCTCT**TCGTCGGCAGCGTC
S505 i5	AATGATACGGCGACCACCGAGATCTACAC**GTAAGGAG**TCGTCGGCAGCGTC
S506 i5	AATGATACGGCGACCACCGAGATCTACAC**ACTGCATAT**CGTCGGCAGCGTC
S507 i5	AATGATACGGCGACCACCGAGATCTACAC**AAGGAGTA**TCGTCGGCAGCGTC
S508 i5	AATGATACGGCGACCACCGAGATCTACAC**CTAAGCCT**TCGTCGGCAGCGTC
S510 i5	AATGATACGGCGACCACCGAGATCTACAC**CGTCTAAT**TCGTCGGCAGCGTC
S511 i5	AATGATACGGCGACCACCGAGATCTACAC**TCTCTCCG**TCGTCGGCAGCGTC
S513 i5	AATGATACGGCGACCACCGAGATCTACAC**TCGACTAG**TCGTCGGCAGCGTC
S515 i5	AATGATACGGCGACCACCGAGATCTACAC**TTCTAGCT**TCGTCGGCAGCGTC
S516 i5	AATGATACGGCGACCACCGAGATCTACAC**CCTAGAGT**TCGTCGGCAGCGTC
S517 i5	AATGATACGGCGACCACCGAGATCTACAC**GCGTAAGA**TCGTCGGCAGCGTC
S518 i5	AATGATACGGCGACCACCGAGATCTACAC**CTATTAAG**TCGTCGGCAGCGTC
S520 i5	AATGATACGGCGACCACCGAGATCTACAC**AAGGCTAT**TCGTCGGCAGCGTC
S521 i5	AATGATACGGCGACCACCGAGATCTACAC**GAGCCTTA**TCGTCGGCAGCGTC
S522 i5	AATGATACGGCGACCACCGAGATCTACAC**TTATGCGA**TCGTCGGCAGCGTC
N701 i7	CAAGCAGAAGACGGCATACGAGAT**TCGCCTTA**GTCTCGTGGGCTCGG
N702 i7	CAAGCAGAAGACGGCATACGAGAT**CTAGTACG**GTCTCGTGGGCTCGG
N703 i7	CAAGCAGAAGACGGCATACGAGAT**TTCTGCCT**GTCTCGTGGGCTCGG
N704 i7	CAAGCAGAAGACGGCATACGAGAT**GCTCAGGA**GTCTCGTGGGCTCGG
N705 i7	CAAGCAGAAGACGGCATACGAGAT**AGGAGTCC**GTCTCGTGGGCTCGG
N706 i7	CAAGCAGAAGACGGCATACGAGAT**CATGCCTA**GTCTCGTGGGCTCGG
N707 i7	CAAGCAGAAGACGGCATACGAGAT**GTAGAGAG**GTCTCGTGGGCTCGG
N708 i7	CAAGCAGAAGACGGCATACGAGAT**CCTCTCTG**GTCTCGTGGGCTCGG
N709 i7	CAAGCAGAAGACGGCATACGAGAT**AGCGTAGC**GTCTCGTGGGCTCGG
N710 i7	CAAGCAGAAGACGGCATACGAGAT**CAGCCTCG**GTCTCGTGGGCTCGG
N711 i7	CAAGCAGAAGACGGCATACGAGAT**TGCCTCTT**GTCTCGTGGGCTCGG
N712 i7	CAAGCAGAAGACGGCATACGAGAT**TCCTCTAC**GTCTCGTGGGCTCGG
N714 i7	CAAGCAGAAGACGGCATACGAGAT**TCATGAGC**GTCTCGTGGGCTCGG
N715 i7	CAAGCAGAAGACGGCATACGAGAT**CCTGAGAT**GTCTCGTGGGCTCGG
N716 i7	CAAGCAGAAGACGGCATACGAGAT**TAGCGAGT**GTCTCGTGGGCTCGG
N718 i7	CAAGCAGAAGACGGCATACGAGAT**GTAGCTCC**GTCTCGTGGGCTCGG
N719 i7	CAAGCAGAAGACGGCATACGAGAT**TACTACGC**GTCTCGTGGGCTCGG
N720 i7	CAAGCAGAAGACGGCATACGAGAT**AGGCTCCG**GTCTCGTGGGCTCGG
N721 i7	CAAGCAGAAGACGGCATACGAGAT**GCAGCGTA**GTCTCGTGGGCTCGG
N722 i7	CAAGCAGAAGACGGCATACGAGAT**CTGCGCAT**GTCTCGTGGGCTCGG
N723 i7	CAAGCAGAAGACGGCATACGAGAT**GAGCGCTA**GTCTCGTGGGCTCGG
N724 i7	CAAGCAGAAGACGGCATACGAGAT**CGCTCAGT**GTCTCGTGGGCTCGG
N726 i7	CAAGCAGAAGACGGCATACGAGAT**GTCTTAGG**GTCTCGTGGGCTCGG
N727 i7	CAAGCAGAAGACGGCATACGAGAT**ACTGATCG**GTCTCGTGGGCTCGG
N728 i7	CAAGCAGAAGACGGCATACGAGAT**TAGCTGCA**GTCTCGTGGGCTCGG
N729 i7	CAAGCAGAAGACGGCATACGAGAT**GACGTCGA**GTCTCGTGGGCTCGG
