# Supplementary material for: Characterization of the β-tubulin gene family in Ascaris lumbricoides and Ascaris suum and its implication for the molecular detection of benzimidazole resistance
Source: PLoS Negl Trop Dis. 2021 Sep 27;15(9):e0009777. doi: 10.1371/journal.pntd.0009777 (PMC8496844; doi:10.1371/journal.pntd.0009777)
Supplement: S5 Info — Alignment of amino acid sequences of the identified β-tubulins of both A. suum and A. lumbricoides. For aesthetic reasons, all sequences were trimmed to a uniform length of 427 amino acids. (N-terminus trimmed: Asu-bt-B, Asu-bt-E; C-terminus trimmed: all). (PDF) [file pntd.0009777.s005.pdf]

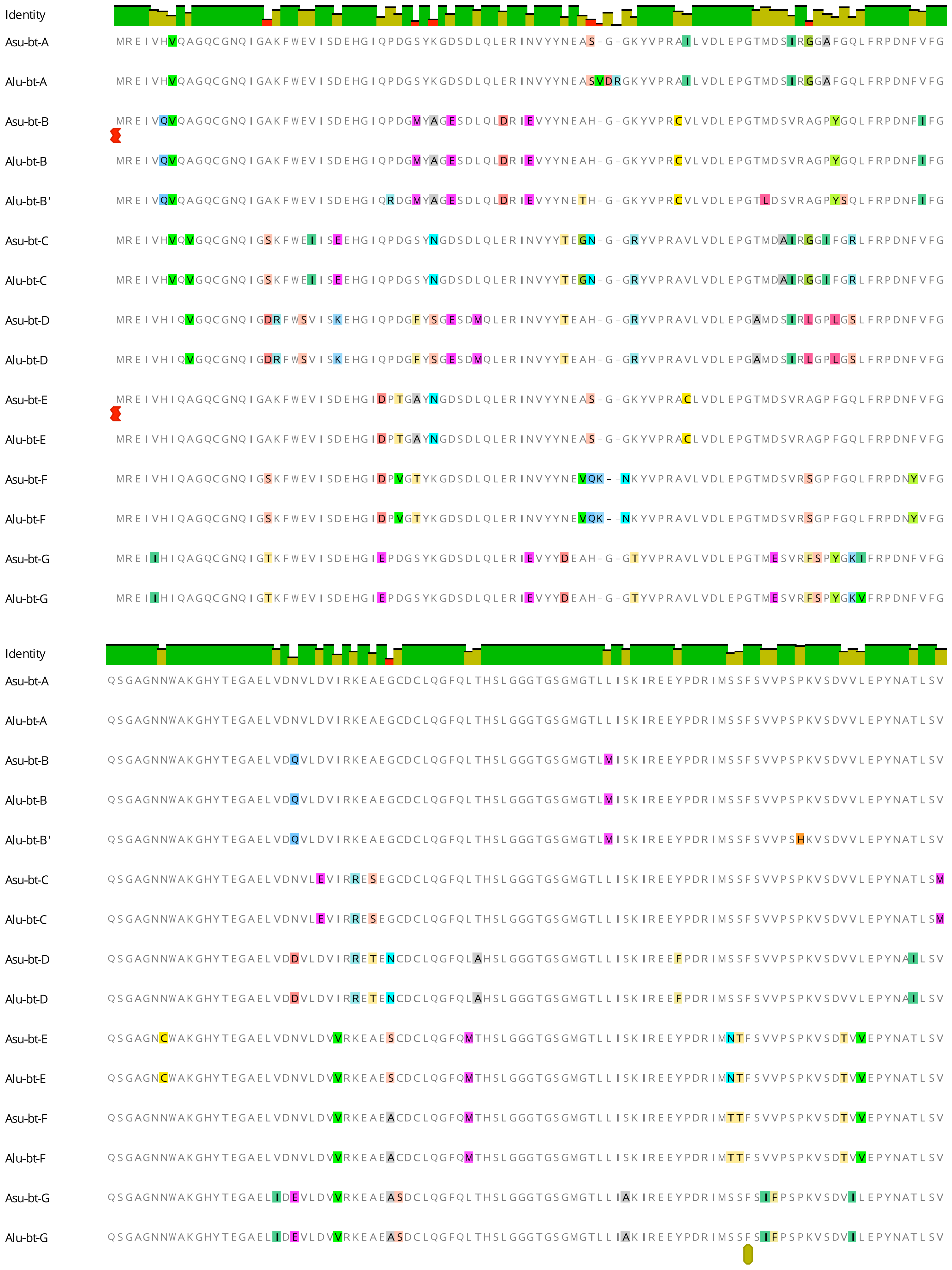
Identity
Asu-bt-A MREIVHVQAGQCGNQIGAKFWEVISDEHGIQPDGSYKGDSDLQLERINVYYNEAS-G-GKYVPRAILVDLEPGTMDSIRGGAFGQLFRPDNFVFG
Alu-bt-A MREIVHVQAGQCGNQIGAKFWEVISDEHGIQPDGSYKGDSDLQLERINVYYNEASVDRGKYVPRAILVDLEPGTMDSIRGGAFGQLFRPDNFVFG
Asu-bt-B MREIVQVQAGQCGNQIGAKFWEVISDEHGIQPDGMYAGESDLQLDRIEVYYNEAH-G-GKYVPRCVLVDLEPGTMDSVRAGPYGQLFRPDNFIFG
Alu-bt-B MREIVQVQAGQCGNQIGAKFWEVISDEHGIQPDGMYAGESDLQLDRIEVYYNEAH-G-GKYVPRCVLVDLEPGTMDSVRAGPYGQLFRPDNFIFG
Alu-bt-B' MREIVQVQAGQCGNQIGAKFWEVISDEHGIQRDGMYAGESDLQLDRIEVYYNETH-G-GKYVPRCVLVDLEPGTLDSVRAGPYSQLFRPDNFIFG
Asu-bt-C MREIVHVQVGQCGNQIGSKFWEIISEEHGIQPDGSYNGDSDLQLERINVYYTEGN-G-GRYVPRAVLVDLEPGTMDAIRGGIFGRLFRPDNFVFG
Alu-bt-C MREIVHVQVGQCGNQIGSKFWEIISEEHGIQPDGSYNGDSDLQLERINVYYTEGN-G-GRYVPRAVLVDLEPGTMDAIRGGIFGRLFRPDNFVFG
Asu-bt-D MREIVHIQVGQCGNQIGDRFWSVISKEHGIQPDGFYSGESDMQLERINVYYTEAH-G-GRYVPRAVLVDLEPGAMDSIRLGPLGSLFRPDNFVFG
Alu-bt-D MREIVHIQVGQCGNQIGDRFWSVISKEHGIQPDGFYSGESDMQLERINVYYTEAH-G-GRYVPRAVLVDLEPGAMDSIRLGPLGSLFRPDNFVFG
Asu-bt-E MREIVHIQAGQCGNQIGAKFWEVISDEHGIDPTGAYNGDSDLQLERINVYYNEAS-G-GKYVPRACLVDLEPGTMDSVRAGPFGQLFRPDNFVFG
Alu-bt-E MREIVHIQAGQCGNQIGAKFWEVISDEHGIDPTGAYNGDSDLQLERINVYYNEAS-G-GKYVPRACLVDLEPGTMDSVRAGPFGQLFRPDNFVFG
Asu-bt-F MREIVHIQAGQCGNQIGSKFWEVISDEHGIDPVGTYKGDSDLQLERINVYYNEVQK–NKYVPRAVLVDLEPGTMDSVRSGPFGQLFRPDNYVFG
Alu-bt-F MREIVHIQAGQCGNQIGSKFWEVISDEHGIDPVGTYKGDSDLQLERINVYYNEVQK–NKYVPRAVLVDLEPGTMDSVRSGPFGQLFRPDNYVFG
Asu-bt-G MREIIHIQAGQCGNQIGTKFWEVISDEHGIEPDGSYKGDSDLQLERIEVYYDEAH-G-GTYVPRAVLVDLEPGTMESVRFSPYGKIFRPDNFVFG
Alu-bt-G MREIIHIQAGQCGNQIGTKFWEVISDEHGIEPDGSYKGDSDLQLERIEVYYDEAH-G-GTYVPRAVLVDLEPGTMESVRFSPYGKVFRPDNFVFG
Identity
Asu-bt-A QSGAGNNWAKGHYTEGAELVDNVLDVIRKEAEGCDCLQGFQLTHSLGGGTGSGMGTLLISKIREEYPDRIMSSFSVVPSPKVSDVVLEPYNATLSV
Alu-bt-A QSGAGNNWAKGHYTEGAELVDNVLDVIRKEAEGCDCLQGFQLTHSLGGGTGSGMGTLLISKIREEYPDRIMSSFSVVPSPKVSDVVLEPYNATLSV
Asu-bt-B QSGAGNNWAKGHYTEGAELVDQVLDVIRKEAEGCDCLQGFQLTHSLGGGTGSGMGTLMISKIREEYPDRIMSSFSVVPSPKVSDVVLEPYNATLSV
Alu-bt-B QSGAGNNWAKGHYTEGAELVDQVLDVIRKEAEGCDCLQGFQLTHSLGGGTGSGMGTLMISKIREEYPDRIMSSFSVVPSPKVSDVVLEPYNATLSV
Alu-bt-B' QSGAGNNWAKGHYTEGAELVDQVLDVIRKEAEGCDCLQGFQLTHSLGGGTGSGMGTLMISKIREEYPDRIMSSFSVVPSHKVSDVVLEPYNATLSV
Asu-bt-C QSGAGNNWAKGHYTEGAELVDNVLEVIRRESEGCDCLQGFQLTHSLGGGTGSGMGTLLISKIREEYPDRIMSSFSVVPSPKVSDVVLEPYNATLSM
Alu-bt-C QSGAGNNWAKGHYTEGAELVDNVLEVIRRESEGCDCLQGFQLTHSLGGGTGSGMGTLLISKIREEYPDRIMSSFSVVPSPKVSDVVLEPYNATLSM
Asu-bt-D QSGAGNNWAKGHYTEGAELVDDVLDVIRRETENCDCLQGFQLAHSLGGGTGSGMGTLLISKIREEFPDRIMSSFSVVPSPKVSDVVLEPYNAILSV
Alu-bt-D QSGAGNNWAKGHYTEGAELVDDVLDVIRRETENCDCLQGFQLAHSLGGGTGSGMGTLLISKIREEFPDRIMSSFSVVPSPKVSDVVLEPYNAILSV
Asu-bt-E QSGAGNCWAKGHYTEGAELVDNVLDVVRKEAESCDCLQGFQMTHSLGGGTGSGMGTLLISKIREEYPDRIMNTFSVVPSPKVSDTVVEPYNATLSV
Alu-bt-E QSGAGNCWAKGHYTEGAELVDNVLDVVRKEAESCDCLQGFQMTHSLGGGTGSGMGTLLISKIREEYPDRIMNTFSVVPSPKVSDTVVEPYNATLSV
Asu-bt-F QSGAGNNWAKGHYTEGAELVDNVLDVVRKEAEACDCLQGFQMTHSLGGGTGSGMGTLLISKIREEYPDRIMTTFSVVPSPKVSDTVVEPYNATLSV
Alu-bt-F QSGAGNNWAKGHYTEGAELVDNVLDVVRKEAEACDCLQGFQMTHSLGGGTGSGMGTLLISKIREEYPDRIMTTFSVVPSPKVSDTVVEPYNATLSV
Asu-bt-G QSGAGNNWAKGHYTEGAELIDEVLDVVRKEAEASDCLQGFQLTHSLGGGTGSGMGTLLIAKIREEYPDRIMSSFSIFPSPKVSDVILEPYNATLSV
Alu-bt-G QSGAGNNWAKGHYTEGAELIDEVLDVVRKEAEASDCLQGFQLTHSLGGGTGSGMGTLLIAKIREEYPDRIMSSFSIFPSPKVSDVILEPYNATLSV

```
Identity
Asu-bt-A   HQLVENTDETFCIDNEALYDICFRTLKLTNPTYGDLNHLVSVTMSGVTTCLRFPGQLNADLRKLAVNMVPFPRLHFFMPGFAPLSARGAAAYRALN
Alu-bt-A   HQLVENTDETFCIDNEALYDICFRTLKLTNPTYGDLNHLVSVTMSGVTTCLRFPGQLNADLRKLAVNMVPFPRLHFFMPGFAPLSARGAAAYRALN
Asu-bt-B   HQLVENTDETFCIDNEALYDICFRTLKLTNPSYGDLNHLVSMTMSGVTTCLRFPGQLNADLRKLAVNMVPFPRLHFFMPGFAPLSARGTAAYRALT
Alu-bt-B   HQLVENTDETFCIDNEALYDICFRTLKLTNPSYGDLNHLVSMTMSGVTTCLRFPGQLNADLRKLAVNMVPFPRLHFFMPGFAPLSARGTAAYRALT
Alu-bt-B'  HELIENTNETFCIDNEALYDICFRTLKLTNPNYGDLNHLVSMTMSGVTTCLRFPGQLNADLRKLAVNMVPFPRLHFFMPGFAPLSARGTAAYRALT
Asu-bt-C   HQLVENTDETFCIDNEALYDICLRTLKLSNPAYGDLNHLVSLTMSGVTTCLRFPGQLNADLRKLAVNMVPFPRLHFFVPGFAPLCARGSDAYEAHN
Alu-bt-C   HQLVENTDETFCIDNEALYDICLRTLKLSNPAYGDLNHLVSLTMSGVTTCLRFPGQLNADLRKLAVNMVPFPRLHFFVPGFAPLCARGSDAYEAHN
Asu-bt-D   HQLLENTDETYCIDNEALYNICFRTLKLSSPSYRDLNNLVSMTMSGVTTCLRFPGQLNADLRKLAVNMVPFPRLHFFVPSFAPLCSRGAVAFQALN
Alu-bt-D   HQLLENTDETYCIDNEALYNICFRTLKLSSPSYRDLNNLVSMTMSGVTTCLRFPGQLNADLRKLAVNMVPFPRLHFFVPSFAPLCSRGAVAFQALN
Asu-bt-E   HQLVENTDETFCIDNEALYDICFRTLKLTTPTYGDLNHLVSMTMSGVTTCLRFPGQLNADLRKLAVNMVPFPRLHFFMPGFAPLTSRGSQQYRSLT
Alu-bt-E   HQLVENTDETFCIDNEALYDICFRTLKLTTPTYGDLNHLVSMTMSGVTTCLRFPGQLNADLRKLAVNMVPFPRLHFFMPGFAPLTSRGSQQYRSLT
Asu-bt-F   HQLVENTDETFCIDNEALYDICFRTLKLTTPTYGDLNHLVSATMSGVTTCLRFPGQLNADLRKLAVNMVPFPRLHFFMPGFAPLTSRSNQQYRAIT
Alu-bt-F   HQLVENTDETFCIDNEALYDICFRTLKLTTPTYGDLNHLVSATMSGVTTCLRFPGQLNADLRKLAVNMVPFPRLHFFMPGFAPLTSRSNQQYRAIT
Asu-bt-G   HQLVENTDETFCIDNEALYDICLRTLKLTGPTYGDLNHLVSMAMSGVTTCLRFPGQLNADLRKLAVNMIPFPRLHFFMPGFAPLTARGAAAYRSLT
Alu-bt-G   HQLVENTDETFCIDNEALYDICLRTLKLTGPTYGDLNHLVSMAMSGVTTCLRFPGQLNADLRKLAVNMIPFPRLHFFMPGFAPLTARGAAAYRSLT

Identity
Asu-bt-A   VAELTQQMFDAKNMMAACDPRHGRYLTVAAMFRGRMSMREVDEQMMQVQNKNSSYFVEWIPNNVKTAVCDIPPRGLKMSATFIGNTTAIQELFKRI
Alu-bt-A   VAELTQQMFDAKNMMAACDPRHGRYLTVAAMFRGRMSMREVDEQMMQVQNKNSSYFVEWIPNNVKTAVCDIPPRGLKMSATFIGNTTAIQELFKRI
Asu-bt-B   VAELTQQVFDAKNMMAACDPRHGRYLTVAVMFRGRMSMREVDNQMLNVQNKNSSYFVEWIPNNVKTAVCDIPPRGLKMSATFMGNTTAIQELFRRI
Alu-bt-B   VAELTQQVFDAKNMMAACDPRHGRYLTVAVMFRGRMSMREVDNQMLNVQNKNSSYFVEWIPNNVKTAVCDIPPRGLKMSATFMGNTTAIQELFRRI
Alu-bt-B'  VAELTQQVFDAKNMMAACDPRHGRYLTAAVMFRGRMSMREVDDQMLNVQNTNSSYFVEWIPNNVKTAVCDIPPRGLKMSATFMGNTTAIQELFRRI
Asu-bt-C   VAELTRQIFDAKNTMAACDPRHGRYLTVAAIFRGRMSMREVDDQMMQVQNKNSTYFVDWIPNNVKTAVCDIPPRGLKMSATFIGNTTAIQELFRRI
Alu-bt-C   VAELTRQIFDAKNTMAACDPRHGRYLTVAAIFRGRMSMREVDDQMMQVQNKNSTYFVDWIPNNVKTAVCDIPPRGLKMSATFIGNTTAIQELFRRI
Asu-bt-D   VAELTQQMFDAKNMMAACDPHHGRYLTVAAIFRGRISMREVDDHMLKMQNKNSEYFVQWIPHNMKTAVCDIPPHGLKMSATFIGNTTAVQDIFKRL
Alu-bt-D   VAELTQQMFDAKNMMAACDPHHGRYLTVAAIFRGRISMREVDDHMLKMQNKNSEYFVQWIPHNMKTAVCDIPPHGLKMSATFIGNTTAVQDIFKRL
Asu-bt-E   VPELTQQMFDAKNMMAACDPRHGRYLTVAAIFRGRMSMKEVDEQMLNVQNKNSSYFVEWIPNNVKTAVCDIPPRGVKMAATFIGNSTAIQELFKRV
Alu-bt-E   VPELTQQMFDAKNMMAACDPRHGRYLTVAAIFRGRMSMKEVDEQMLNVQNKNSSYFVEWIPNNVKTAVCDIPPRGVKMAATFIGNSTAIQELFKRV
Asu-bt-F   VPELTQQMFDAKNMMAACDPRHGRYLTAAAIFRGRMSMKEVDEQMLNIQNKNSSYFVDWIPNNVKTAVCDIPPRGLRMAATFIGNSTAIQELFKRI
Alu-bt-F   VPELTQQMFDAKNMMAACDPRHGRYLTAAAIFRGRMSMKEVDEQMLNIQNKNSSYFVDWIPNNVKTAVCDIPPRGLRMAATFIGNSTAIQELFKRI
Asu-bt-G   INELIQQLFDSKNMMAACDPRHGRYLTVAAMFRGRLSMHELDMQILNVQNKNSSYFVEWIPNNVKTAVCDIPPRGLKMSATFLGNTTAIQEIFIRI
Alu-bt-G   INELIQQLFDSKNMMAACDPRHGRYLTVAAMFRGRLSMHELDMQILNVQNKNSSYFVEWIPNNVKTAVCDIPPRGLKMSATFLGNTTAIQEIFIRI
```

Identity

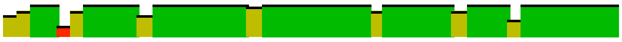

Asu-bt-A	SEQFTAMFRRKAFLHWYTGEGMDEMEFTEAESNMNDLVSEYQQYQ
Alu-bt-A	SEQFTAMFRRKAFLHWYTGEGMDEMEFTEAESNMNDLVSEYQQYQ
Asu-bt-B	SEQFNAMFRRKAFLHWYTGEGMDEMEFTEAESNMNDLISEYQQYQ
Alu-bt-B	SEQFNAMFRRKAFLHWYTGEGMDEMEFTEAESNMNDLISEYQQYQ
Alu-bt-B'	SEQFNAMFRRKAFLHWYTDEGMDEMEFTEAESNMNDLISEYQQYQ
Asu-bt-C	SEQFAAMFRRKAFLHWYTGEGMDEMEFTEAESNMNDLISEYQQYQ
Alu-bt-C	SEQFAAMFRRKAFLHWYTGEGMDEMEFTEAESNMNDLISEYQQYQ
Asu-bt-D	ADQFALMFRRRAFLHWYTGEGMDEMEFIEAESNINDLISEYQQYQ
Alu-bt-D	ADQFALMFRRRAFLHWYTGEGMDEMEFIEAESNINDLISEYQQYQ
Asu-bt-E	SEQFTAMFRRKAFLHWYTGEGMDEMEFTEAESNMNDLISEYQQYQ
Alu-bt-E	SEQFTAMFRRKAFLHWYTGEGMDEMEFTEAESNMNDLISEYQQYQ
Asu-bt-F	SEQFTAMFRRKAFLHWYTGEGMDEMEFTEAESNMNDLVSEYQQYQ
Alu-bt-F	SEQFTAMFRRKAFLHWYTGEGMDEMEFTEAESNMNDLVSEYQQYQ
Asu-bt-G	AEQFEAMFRRRAFLHWYTGEGMDEMEFTEAESNMNDLVSEYQQYQ
Alu-bt-G	AEQFEAMFRRRAFLHWYTGEGMDEMEFTEAESNMNDLVSEYQQYQ
