# Supplementary figures and images for: Characterization of the β-tubulin gene family in Ascaris lumbricoides and Ascaris suum and its implication for the molecular detection of benzimidazole resistance
Source: PLoS Negl Trop Dis. 2021 Sep 27;15(9):e0009777. doi: 10.1371/journal.pntd.0009777 (PMC8496844; doi:10.1371/journal.pntd.0009777)

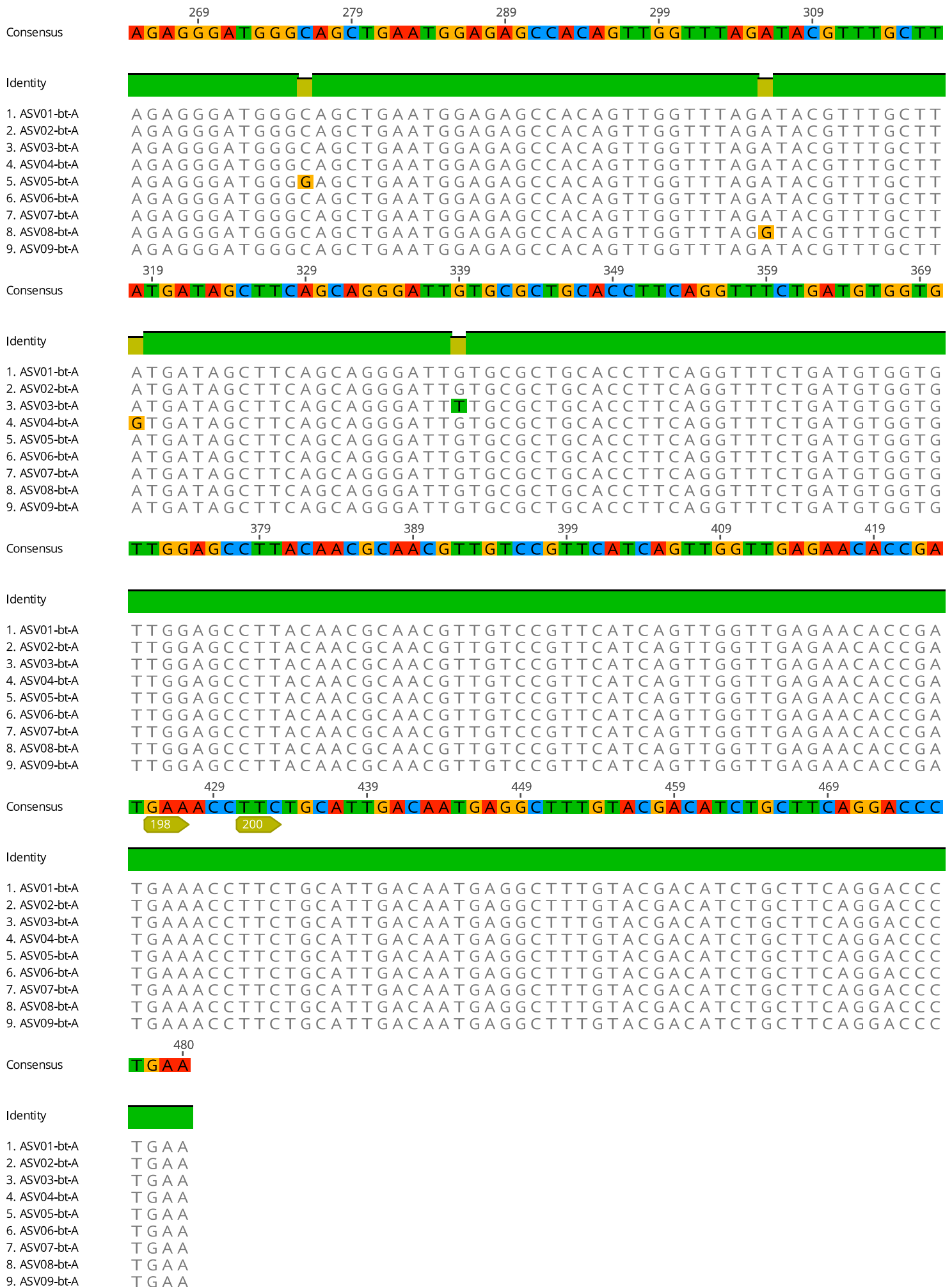

Supplement: S8 Info — Nucleotide alignment of the nine identified amplicon sequence variants (ASVs) of Alu/Asu-bt-A. Multiple alignment was performed using Geneious v10.2.6. The three codons of interest in the context of benzimidazole resistance are indicated by their number at the base of the consensus sequence. (PDF) [file pntd.0009777.s008.pdf]

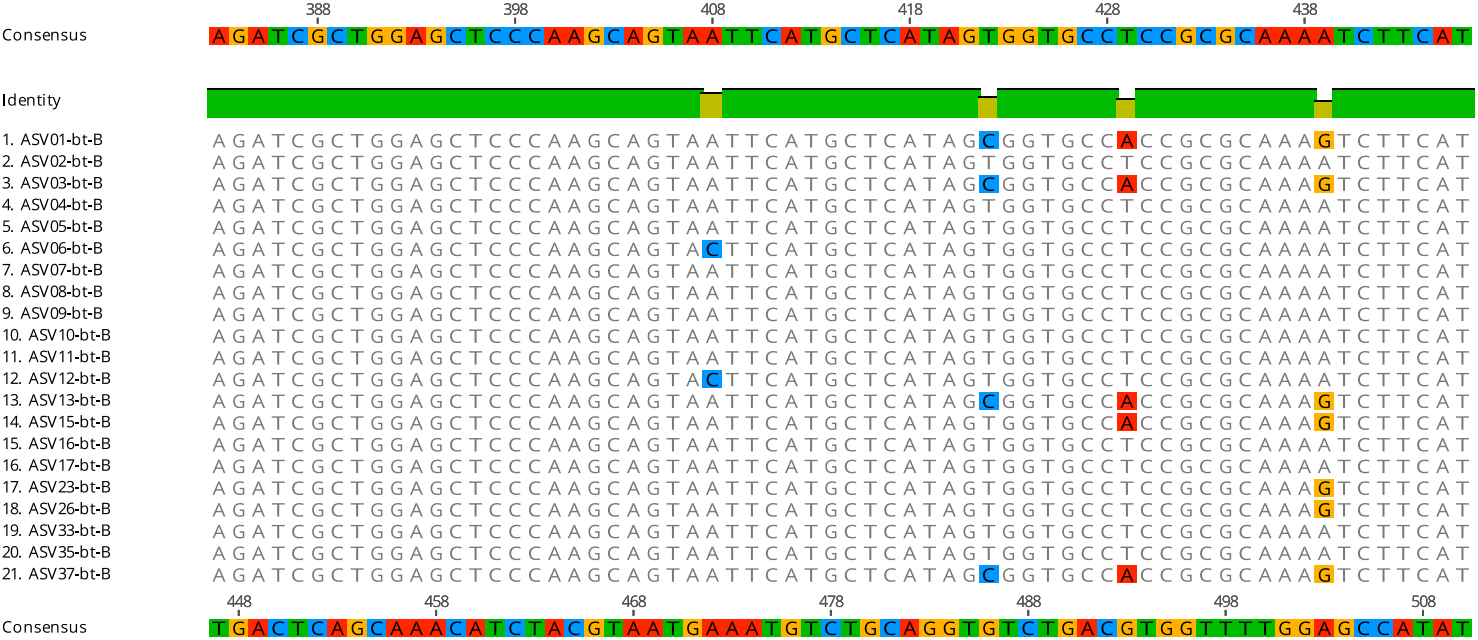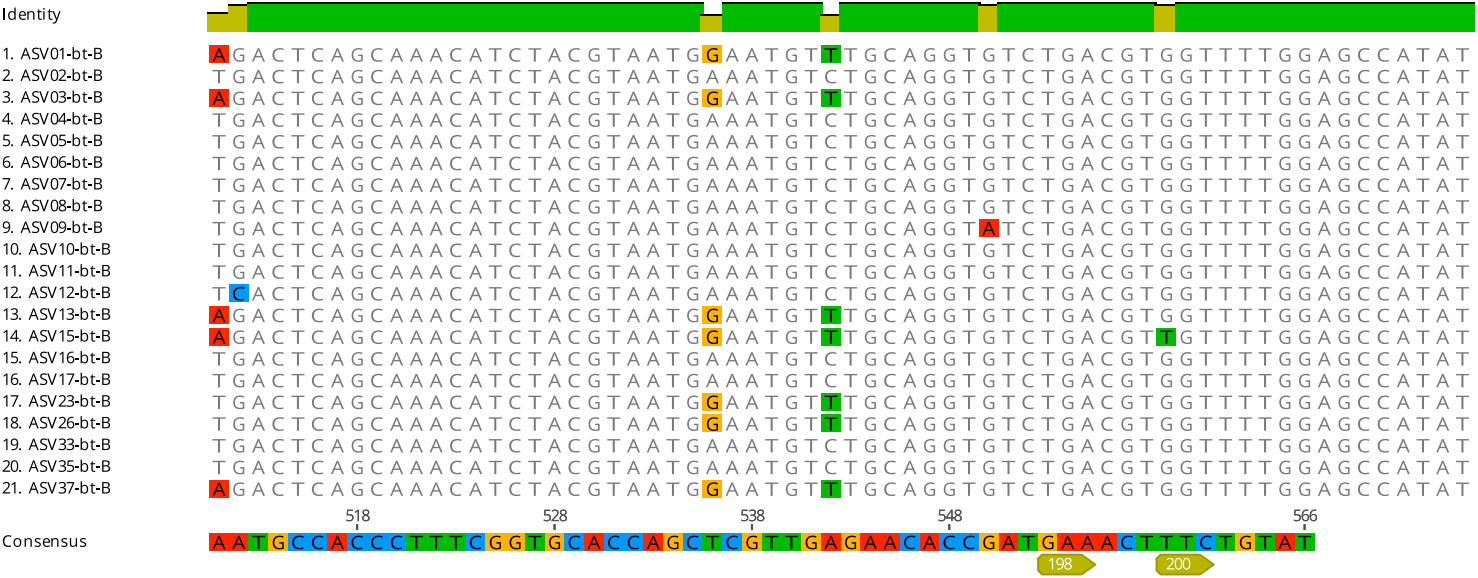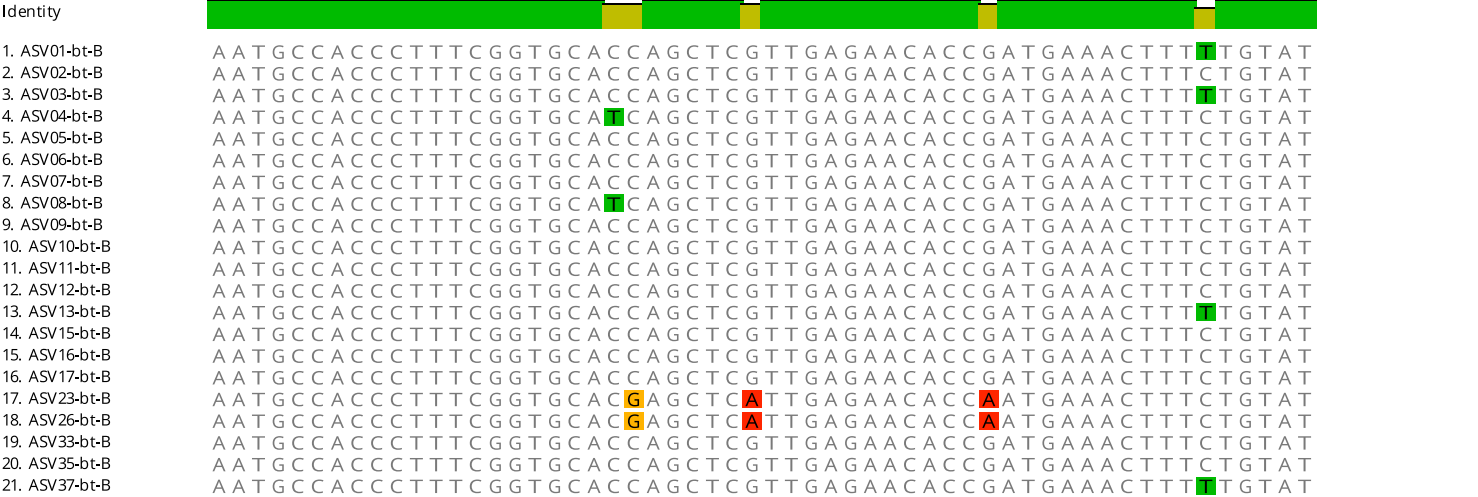

Supplement: S13 Info — Nucleotide alignment of the 21 identified amplicon sequence variants (ASVs) of Alu/Asu-bt-B. Multiple alignment was performed using Geneious v10.2.6. The three codons of interest in the context of benzimidazole resistance are indicated by their number at the base of the consensus sequence. (PDF) [file pntd.0009777.s013.pdf]

Tree scale: 0.1

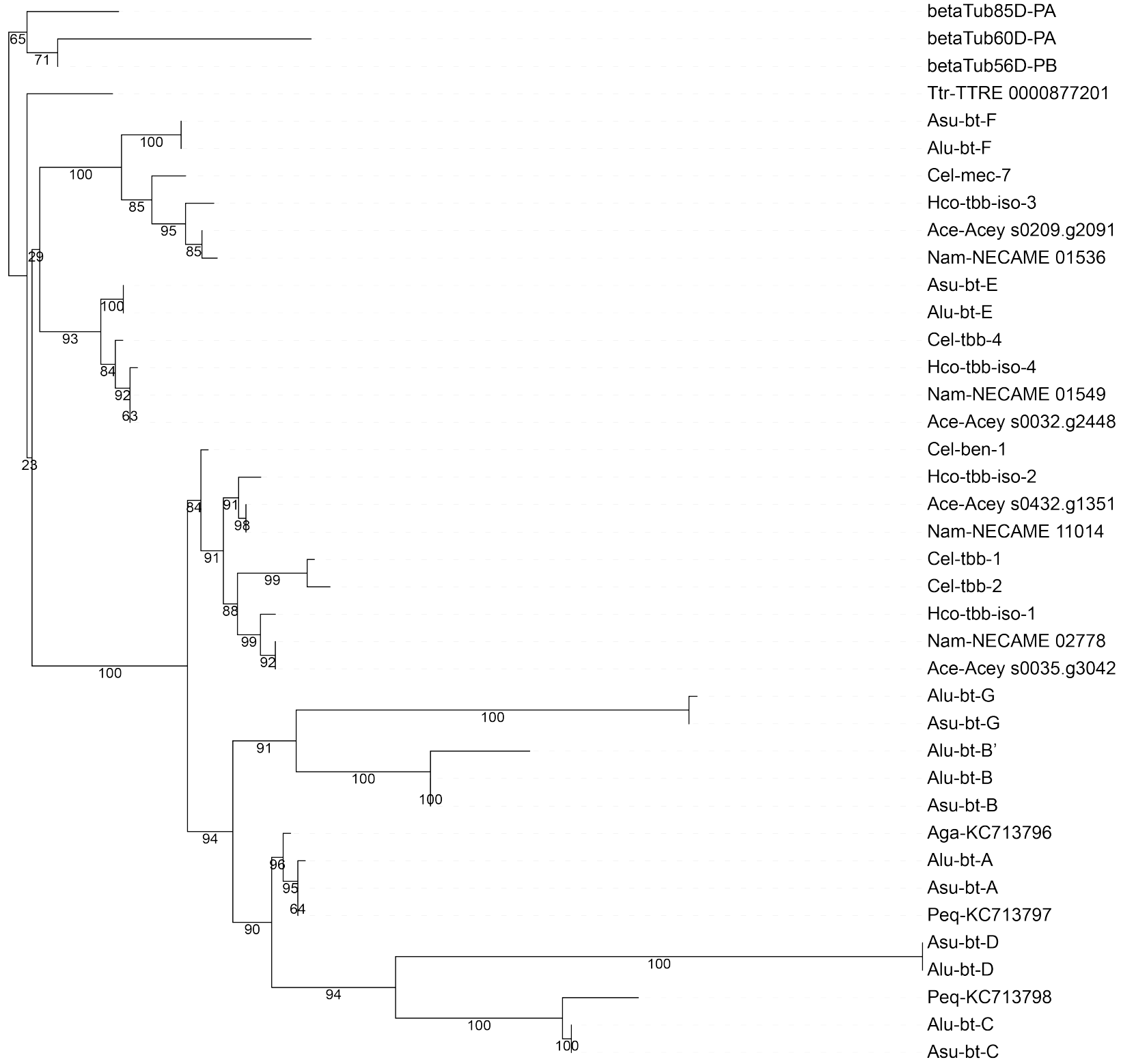

Supplement: S14 Info — Phylogenies were reconstructed with RAxML and MRBAYES. The topology shown is from RAxML. The node support values are percent bootstraps / posterior probabilities. The ‘-‘ indicates that a node was not present in the MRBAYES tree. The branch lengths show divergence. (PDF) [file pntd.0009777.s014.pdf]
